# Evaluating the Impact of Functional Genetic Variation on HIV-1 Control

**DOI:** 10.1093/infdis/jix470

**Published:** 2017-09-09

**Authors:** Paul J McLaren, Sara L Pulit, Deepti Gurdasani, Istvan Bartha, Patrick R Shea, Cristina Pomilla, Namrata Gupta, Effrossyni Gkrania-Klotsas, Elizabeth H Young, Norbert Bannert, Julia Del Amo, M John Gill, Jill Gilmour, Paul Kellam, Anthony D Kelleher, Anders Sönnerborg, Steven M Wolinsky, Robert Zangerle, Frank A Post, Martin Fisher, David W Haas, Bruce D Walker, Kholoud Porter, David B Goldstein, Manjinder S Sandhu, Paul I W de Bakker, Jacques Fellay

**Affiliations:** 1 JC Wilt Infectious Diseases Research Centre, National HIV and Retrovirology Laboratory, Public Health Agency of Canada; 2 Department of Medical Microbiology and Infectious Diseases, University of Manitoba, Winnipeg, Canada; 3 Department of Genetics, Center for Molecular Medicine, University Medical Center Utrecht, The Netherlands; 4 Human Genetics, Wellcome Trust Sanger Institute, Hinxton, United Kingdom; 5 Department of Medicine, University of Cambridge, United Kingdom; 6 Global Health Institute, School of Life Sciences, École Polytechnique Fédérale de Lausanne, Switzerland; 7 Institute for Genomic Medicine, Columbia University, New York; 8 Program in Medical and Population Genetics, Broad Institute of MIT and Harvard, Cambridge, USA; 9 Department of Infectious Diseases, University of Cambridge, United Kingdom; 10 Division of HIV and Other Retroviruses, Robert Koch Institute, Berlin, Germany; 11 Centro Nacional de Epidemiología, Instituto de Salud Carlos III, Madrid, Spain; 12 Department of Medicine, University of Calgary, Canada; 13 Human Immunology Laboratory, International AIDS Vaccine Initiative, Imperial College, London, United Kingdom; 14 Research Department of Infection, Division of Infection and Immunity, University College London, United Kingdom; 15 The Kirby Institute for Infection and Immunity in Society, University of New South Wales, Sydney, Australia; 16 Unit of Infectious Diseases, Department of Medicine Huddinge, Karolinska Institutet, Stockholm, Sweden; 17 Division of Infectious Diseases, The Feinberg School of Medicine, Northwestern University, Chicago; 18 Department of Dermatology and Venereology, Medical University Innsbruck, Austria; 19 Kings College Hospital, London, United Kingdom; 20 Royal Sussex County Hospital, Brighton, United Kingdom; 21 Department of Medicine, Vanderbilt University School of Medicine, Nashville; 22 Ragon Institute of Massachusetts General Hospital, Massachusetts Institute of Technology, and Harvard, Boston; 23 Howard Hughes Medical Institute, Chevy Chase; 24 University College London, United Kingdom; 25 Department of Epidemiology, Julius Center for Health Sciences and Primary Care, University Medical Center Utrecht, The Netherlands; 26 Swiss Institute of Bioinformatics, Lausanne, Switzerland

**Keywords:** HIV-1 control, exome sequencing, HIV-1 progression, host genetics of infection, HIV host dependency factors

## Abstract

**Background:**

Previous genetic association studies of human immunodeficiency virus-1 (HIV-1) progression have focused on common human genetic variation ascertained through genome-wide genotyping.

**Methods:**

We sought to systematically assess the full spectrum of functional variation in protein coding gene regions on HIV-1 progression through exome sequencing of 1327 individuals. Genetic variants were tested individually and in aggregate across genes and gene sets for an influence on HIV-1 viral load.

**Results:**

Multiple single variants within the major histocompatibility complex (MHC) region were observed to be strongly associated with HIV-1 outcome, consistent with the known impact of classical HLA alleles. However, no single variant or gene located outside of the MHC region was significantly associated with HIV progression. Set-based association testing focusing on genes identified as being essential for HIV replication in genome-wide small interfering RNA (siRNA) and clustered regularly interspaced short palindromic repeats (CRISPR) studies did not reveal any novel associations.

**Conclusions:**

These results suggest that exonic variants with large effect sizes are unlikely to have a major contribution to host control of HIV infection.

## Teaser

We evaluated the impact of coding variation on HIV control by exome sequencing with emphasis on known HIV host dependency factors. Outside of the MHC region, exonic variants with large effect sizes are not a major contributor to HIV control.

Controlling human immunodeficiency virus-1 (HIV-1) viral load in infected patients is necessary to reduce global morbidity, mortality, and viral transmission [1]. Given the difficulties in developing an effective anti-HIV vaccine and the lack of efficient strategies to eradicate the virus in infected individuals, current and novel drug classes will be required for long-term viral suppression and global HIV prevention. Advances in understanding of the scope of functional human genetic variation and its impact on health and disease provide an attractive strategy for identification of novel drug targets [2]. Such a strategy leverages population genetic variation in key proteins that modify their function, thus honing in on potential novel drug targets. This approach has already been successfully applied in the development of maraviroc, a *CCR5* antagonist that mimics the effect of the highly protective CCR5Δ32 polymorphism [3], which confers resistance to infection in homozygous carriers.

In recent years, genome-wide association studies of HIV control have repeatedly underscored the large impact of common genetic variation ascertained through genome-wide genotyping (minor allele frequency > 5%) [4–6]. These studies have identified multiple independent polymorphisms within the *HLA* and *CCR5* regions that together explain approximately 25% of the observed variability in viral load [7]. However, such studies do not directly assess the impact of the full spectrum of functional variants within coding regions and such polymorphisms, which are not well represented in genome-wide association studies, have been proposed to explain an additional proportion of viral load variability [8, 9]. Indeed, exome sequencing has uncovered functional variants contributing to the genetic architecture of a number of complex traits [10–13]. In this study, we sought to address this gap by combining exome sequencing data from 5 independent studies of HIV progression. After quality control, we obtained high-quality sequence data for 1327 individuals, including 962 people living with HIV that had varying rates of spontaneous viral control and disease progression. Identification of functional variants that impact HIV disease, particularly in genes required for HIV replication identified in genome-wide small interfering RNA (siRNA) [14] and clustered regularly interspaced short palindromic repeats (CRISPR) [15] screens, may help to inform drug development and stem the transmission of the virus.

## MATERIALS AND METHODS

### Sample Cohorts and Sequencing

Written, informed consent for genetic studies was obtained from all participants. Ethical approval for each study was obtained from their local Institutional Review Board. A total of 1003 nonoverlapping, HIV infected individuals were recruited across 5 independent studies comprising 3 separate study designs as follows (and Supplementary Table 1):

1. Quantitative set point viral load (spVL): Patients enrolled in the Swiss HIV Cohort Study (SHCS, n = 392) were selected based on stability of spVL measurement in the absence of antiretroviral therapy (ART). Participants for exome sequencing were selected at random from a larger set of individuals with high-quality set point HIV viral load determinations. All viral load measurements were made off therapy during the chronic phase of infection. Full details of inclusion/exclusion criteria have been described previously [16]. In this group, log_10_(spVL) was used as a quantitative trait for association testing.
2. HIV elite controllers compared to population controls: HIV elite controllers (n = 219), defined as ART-naive individuals having stable spVL measurements below 50 copies per mL of plasma, were enrolled in the International HIV Controllers Study (IHCS) [6]. To enhance discovery at novel loci, controllers were preferentially included if they did not carry known protective *HLA-B* alleles (B*57:01, B*14:02 and B*27:05). Controllers were matched to 64 HIV-infected individuals with high spVL enrolled in ART studies led by the AIDS Clinical Trials Group (ACTG) and 372 HIV-uninfected individuals included as controls in the Autism Sequencing Consortium (ASC) [17], resulting in an initial sample size of 219 cases and 436 controls. Controller or noncontroller/HIV status was used as a binary endpoint for association testing.
3. HIV controllers (HIV-C) compared to rapid progressors (HIV-RP): Patients were recruited to the CASCADE study (n = 85 HIV-C, 98 HIV-RP), the Multicenter AIDS Cohort Study (MACS, n = 34 HIV-C, 54 HIV-RP), or the HIV Genomics Consortium (HGC, n = 21 HIV-C, 36 HIV-RP), resulting in an initial sample size of 140 HIV-C and 188 HIV-RP. HIV-Cs were defined as HIV+ individuals with sustained, suppressed viral loads and/or long-term survival off therapy. HIV-RPs were defined as individuals exhibiting low CD4 counts within 6 months to 3 years postinfection (precise phenotype definitions used per cohort are given in Supplementary Table 1). HIV-C and HIV-RP were used as binary endpoints in a case/control framework for association testing.

Participants predominantly reported European ancestry (Supplementary Table 1), which was confirmed by principal components analysis using EIGENSTRAT [18]. Exon capture was performed using the Illumina Truseq 65Mb enrichment kit (SHCS), Agilent 38Mb SureSelect v2 enrichment kit (IHCS, ACTG, ASC), Agilent SureSelect Human All Exon 37Mb V1 (MACS), or Agilent SureSelect Human All Exon 50Mb v5 (CASCADE and HGC) (Supplementary Table 2). All exons were sequenced to high coverage on either the Illumina HiSeq 2000 or Illumina Genome Analyzer II at local sequencing centers. For all samples, >90% of targeted bases had >10× coverage and >80% of targeted bases had >20× coverage.

### Sequence Alignment and Variant Calling

Paired-end, short read data passing Illumina quality filters were aligned to the human reference genome version 19 (hg19/GhCR37) using the Burrows–Wheeler Aligner [19], polymerase chain reaction (PCR) duplicates were removed using Samtools and quality score recalibration and realignment around insertion/deletion variants (indels) was performed using the Genome Analysis Toolkit version 3.1-1 (GATK). Variant calling of single nucleotide variants (SNVs) and small indels was performed on the combined sample using the HaplotypeCaller module of the GATK, and variant quality score recalibration (VQSR) was done using training sets released with GATK v. 3.1-1 and corresponding best practices. Only variants passing the variant quality score recalibration (VQSR) thresholds were maintained for further analysis. After variant calling, samples were separated by phenotype group as outlined in the previous section.

### Sample and Variant Quality Control

Sample quality was evaluated using a selected set of high frequency (> 5%) SNVs with low missingness (< 2%). For each phenotype group, sample duplicates were detected using identity-by-descent analysis and a single duplicate was removed (n = 11). Samples having high missingness (>5%, n = 19) or abnormal heterozygosity levels (inbreeding coefficient > 0.1 or < −0.1, n = 13) were removed. Specific to the IHCS set, samples with an excessive number of singletons (> 3 standard deviations from the mean of all samples) were also removed (n = 5). A summary of the final sample numbers is provided in Table 1.

**Table 1.** Study Designs and Sample Numbers Included

Study design	Description	n cases	n controls	n total	Phenotype class	Contributors
Natural history	HIV+ patients with spVL selected from full phenotype distribution	na	na	392	quantitative	Swiss HIV Cohort Study
HIV extreme vs population	HIV elite controllers compared to a mixed sample of HIV+ and HIV negative	191 (HIV-EC)	428 (63 HIV+)	619	binary	International HIV Controllers Study, AIDS Clinical Trials Group, Autism Sequence Consortium
HIV extreme	HIV controllers compared to HIV rapid progressors	130 (HIV-C)	186 (HIV-RP)	316	binary	CASCADE, Multicenter AIDS Cohort Study, HIV Genomics Consortium

Abbreviations: HIV-EC, HIV elite controllers; HIV-C, HIV controllers; HIV-RP, HIV rapid progressors; spVL, set point viral load; na, not applicable.

For all phenotype groups, variants were removed if they diverged from Hardy–Weinberg equilibrium (HWE, *P* < 1 × 10^–5^) or were missing in > 5% of individuals. Specific to the IHCS sample, a stricter threshold was applied to account for variants with highly heterozygous calls (HWE *P* < .01). Additionally, all indels were removed from the IHCS set as there was a systematic case/control bias in indel calls resulting in inflation of association results.

### Variant Annotation, Association Testing, and Meta-analysis

Per phenotype group, variants were annotated for functional consequences using SNPeff v3.1. For single variant association testing, we restricted to variants with frequency of >1% and used linear mixed models as implemented in FaST-LMM v2.07 [20] to test for association with either spVL (quantitative trait) or case/control status. Variant *P* values were combined across groups using a sample size weighted, signed Z-score meta-analysis method [21] assuming the same direction of effect for alleles associating with lower spVL and with a higher frequency in HIV controllers compared to rapid progressors or the general population. Multiple comparisons were accounted for using Bonferroni correction and variants with *P* < 9 × 10^–7^ were considered significant. Power for variant detection was calculated using the online Genetic Power calculator [22] assuming a sample size of 1000, a case/control ratio of 1, and a trait prevalence of 0.05. Linkage disequilibrium and haplotype structure between associated SNPs in the MHC region and classical alleles of HLA-A, -B, and -C were calculated using PLINKv1.9 [23] in a subset of 367 individuals from the SHCS set with HLA types imputed previously [7].

### Gene and Gene Set Association Testing

Gene and gene set association testing was performed using the default weighting scheme of SKAT-O as implemented in the R statistical software [24]. This method evaluates the evidence for association across all variants within a gene or gene set, providing additional weight to low-frequency variants. Analysis was performed using all variants and restricting to functional variants (SNPeff category MODERATE) or highly damaging variants (SNPeff category HIGH). Association evidence was combined across groups using Fisher’s method. We used Bonferroni correction assuming 20000 genes (*P* < 2.5 × 10^−6^) to consider a gene or set significantly associated. We used the power simulation function built in to the SKAT library in R to estimate power across a range of scenarios assuming the same sample size as for the single variant analysis.

### Analysis of Variation in HIV Dependency Factors

Gene lists of HIV dependency factors were taken from recent siRNA knockdown [14] and CRISPR [15] studies. Based on the criteria used in the siRNA study, we generated gene sets of HIV dependency factors at two different significance levels (false discovery rate q values of 0.2 and 0.05). In addition, we tested sets including all genes in the mediator complex and the nuclear pore complex, which were identified in the siRNA study as key cellular components required for HIV replication. From the CRISPR study, 4 high-confidence HIV dependency factor genes not identified by the siRNA studies were included. A list of genes included in each set is provided in Supplementary Table 3. Association testing for each HIV dependency factor gene and gene set was performed using SKAT-O under the same framework as the genome-wide screen.

## RESULTS

### Association Testing of Common Polymorphisms

**Figure 1.**
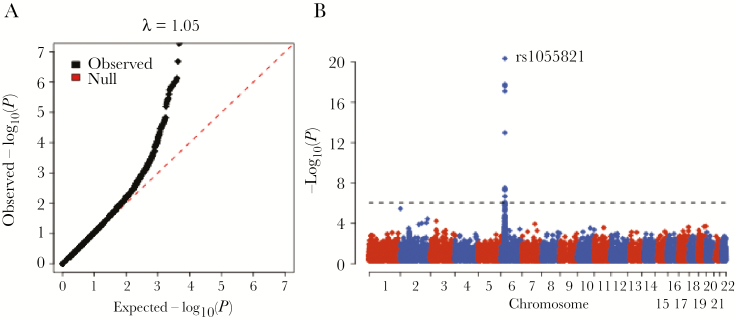
Meta-analysis of association results with HIV load for 55714 variants. *A*, Quantile–quantile plot showing observed −log_10_(*P*) vs expected −log_10_(*P*) under the null hypothesis. The bulk of the observed *P*-value distribution (black diamonds) is in line with the null expectation (dashed red line) and the genomic inflation factor (median observed chi square statistics divided by expected) is approximately 1 (λ = 1.05). This suggests very little inflation in the test statistic dues to confounding factors. Axes are truncated at *P* <1 × 10^−7^. *B*, Manhattan plot. Per variant association *P* values (−log_10_ transformed) are plotted (y axis, colored diamonds) by chromosomal position (x axis). Only variants within the MHC region show significant associations after correcting for multiple testing (*P* <9 × 10^−7^, dashed line).

After quality control, high-quality exome sequence data were available for 962 HIV-infected patients and 365 HIV-uninfected individuals distributed across 3 related study designs (Table 1). In total across all 3 groups, we tested 55714 common (frequency >1%) exonic polymorphisms (SNPs and indels) in 12808 genes for an impact on HIV control. In line with expectation, we observed a strong association between variants in *HLA-B* and HIV phenotypes (Figure 1). The top associated variant, rs1055821 (*P* = 4.6 × 10^–21^), is located in the 3′ untranslated region of *HLA-B* and the minor (A) allele falls on haplotypes containing SNPs previously identified by genome-wide association studies (Supplementary Table 4) as well as HLA-B*57 (r^2^ = 0.60, D′ = 1) and HLA-B*13 (r^2^ = 0.34, D′ = 1), both known to strongly associate with lower HIV viral load [7]. Outside of the MHC region, no variants were significantly associated with HIV phenotypes in the individual group analyses or in the meta-analysis (Supplementary Figure 1).

### Gene-based Association Testing

**Figure 2.**
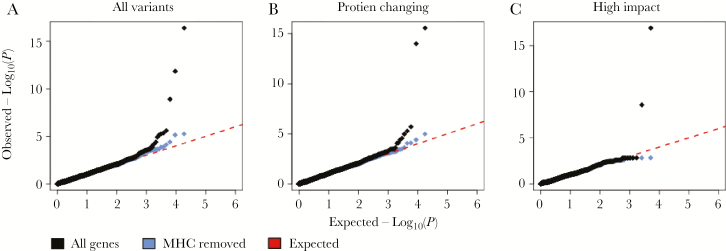
Association results of gene-based testing. Quantile–quantile plots of observed −log_10_(*P*) (diamonds, y axis) vs −log_10_(*P*) expected under the null hypothesis (red dashed line). Analysis was performed including all variants regardless of predicted function (*A*), only variants predicted to modify the protein (*B*), and only variants predicted to highly impact the protein structure (*C*). Only genes within the MHC were associated after correcting for multiple comparisons. The distribution of *P* values of non-MHC genes (blue diamonds) closely follows the expected null distribution.

Detecting the impact of rare functional variants individually on HIV control would require extremely large sample sizes, due to the overall lower frequency (and potentially modest impact) of such variation. To overcome this, we next assessed the evidence that multiple variants (common and rare) within a gene may contribute to HIV control using SKAT-O [24]. Per group, variants were annotated for functional consequences using SNPeff [25] and we tested genes for association with viral load in three iterations: (1) including all variants, (2) restricting to protein-modifying variants (missense, nonsense, frameshift, and splice site), and (3) restricting to predicted loss-of-function variants (nonsense, frameshift, and splice site). As in the single variant analysis, the strongest associations were observed for genes in the MHC region. Outside of the MHC region, no single gene was associated with the HIV control phenotypes under study regardless of the functional consequences of the included variants (Figure 2).

### Assessment of the Impact of Variation Within HIV Dependency Factors

Genetic screens using small interfering RNA (siRNA) molecules [14] or CRIPSR [15] technology to systematically knock out expression of host genes have identified multiple genes required for efficient HIV replication in vitro (termed HIV dependency factors). Although none of these HIV dependency factors were individually identified by gene-based testing, we hypothesized that functional variation within them taken as a set may play a role in control of HIV replication in vivo. To assess this, we generated gene sets combining results from both screens (ie the union) and for each technology separately. From the siRNA screen, we created two sets reflecting different statistical cutoffs for classifying a gene as an HIV dependency factor (false discovery rate < 0.2 and 0.05) consistent with the original publication [14]. In addition, we tested all genes within the mediator complex and the nuclear pore complex given their over-representation in the siRNA studies. Applying the same analysis framework as for genes, we tested each set for association with HIV control. We did not observe a significant association between any set of selected HIV dependency factors and HIV viral load (Table 2). The lists of genes included in each set and their gene-based association results are listed in Supplementary Table 3.

**Table 2.** Set Test Association Results for HIV Dependency Factor Genes

Gene set	n genes	All variants (*P*)	Protein changing variants (*P*)	High impact (*P*)
All HDFs	496	0.08	0.09	0.73
CRISPR	5	0.24	0.10	0.31
siRNA FDR <0.2	492	0.09	0.10	0.75
siRNA FDR <0.05	91	0.38	0.40	0.81
Mediator complex	26	0.47	0.48	0.11
Nuclear pore complex	27	0.56	0.69	0.36

Abbreviations: FDR, false discovery rate; HDFs, HIV dependency factors.

## DISCUSSION

Human genetic variation plays a large role in determining the outcome of HIV-1 infection. However, common, genome-wide genetic factors identified by genome-wide association studies can only explain up to 25% of the observed variability in HIV spVL. To assess the evidence for an additional impact of functional variation in HIV progression, we combined exome sequence data across 5 independent studies that evaluated 3 related models of HIV control in a total of 1327 individuals with high-quality data.

In the single variant analysis, we observed several strongly associated sites, all located in or near the *HLA-B* gene. The top associated SNP, rs1055821, is in strong linkage disequilibrium with classical *HLA-B* alleles known to protect against HIV progression and several other variants previously reported in large, genome-wide association studies (Supplementary Table 4). This suggests that the observed association at this SNP is simply a tag for previously identified functional variants (such as valine at position 97 in the HLA-B peptide binding groove) and that functional variants with large effect sizes not identified by genome-wide association studies are unlikely to play a major role in HIV control. We further assessed the role of an accumulation of variants within a gene for an impact on HIV outcome by using a gene-based association framework. This method has been shown by simulation and in practice to enhance power for genetic discovery, even with relatively small sample sizes [24]. Though we did observe several genes with significant *P* values, each was located within the MHC region and likely a result of the complex linkage disequilibrium and extended haplotype structure between these genes and class I HLA genes.

We also performed a focused analysis on a high-confidence set of HIV dependency factors and a detailed evaluation of the impact of their genetic variation on disease. Although a previous genetic study based on earlier siRNA screens observed associations between polymorphisms in HIV dependency factors and clinical HIV phenotypes [26], we did not see a similar effect. Further, combining variants across the entire set of high-confidence HIV dependency factors or across genes in cellular complexes implicated by siRNA studies (ie the mediator complex and the nuclear pore complex) did not reveal significant associations. This suggests that functional polymorphisms within these genes does not have a large impact on HIV replication in vivo, although we cannot rule out an impact of such variation on HIV acquisition.

This study design was aimed at directly interrogating the impact of functional genetic variation on HIV-1 control across the frequency spectrum. To identify maximum genetic effects detectable in our study, we preformed power simulations reflecting the combined sample size of n = approximately 1000. In the single variant analysis, we had approximately 99% power to detect variants with 10% allele frequency and an odds ratio of 2.0 or greater assuming a case/control ratio of 1 to 1. However, we had only limited power (approximately 0.2%) to detect rare alleles (<1%) with that same effect size. Similarly, for the gene-based association testing, this study was well powered to detect signals of large effect (power was approximately 80% to detect a gene containing 50% causal variation, no protective variation, and a variant with a maximum odds ratio of 8 at *P* = 1 × 10^–6^; Supplementary Figure 2) but not more subtle effects (power was approximately 10% to detect a gene containing 10% causal variation, no protective variation, and a variant with a maximum odds ratio of 8 at *P* = 1 × 10^–6^; Supplementary Figure 2). Thus, we cannot rule out that subtler genetic effects would be detected in a larger study. Additionally, sequencing was restricted to the exonic regions of the genome in a largely European sample. This prevented us from assessing the potential contribution of large structural polymorphisms, rare variants in regulatory regions, and large effect coding variants present in non-European populations. A combination of whole-genome sequencing, in-depth genomic exploration of additional populations from varied ancestral backgrounds in large-scale studies, extension to other clinical phenotypes, and functional in vitro and ex vivo validation studies, performed in step with sequencing of the virus itself, provides an attractive next frontier in HIV host genomics research.

## Supplementary Data

Supplementary materials are available at *The Journal of Infectious Diseases* online. Consisting of data provided by the authors to benefit the reader, the posted materials are not copyedited and are the sole responsibility of the authors, so questions or comments should be addressed to the corresponding author.

## 

***Potential conflicts of interest.*** All authors: No reported conflicts of interest. All authors have submitted the ICMJE Form for Disclosure of Potential Conflicts of Interest. Conflicts that the editors consider relevant to the content of the manuscript have been disclosed.

## Supplementary Material

Supplementary Figure S1

Supplementary Figure S2

Supplementary Table S1

Supplementary Table S2

Supplementary Table S3

Supplementary Table S4

## Notes

***Presented in parts:*** Early versions of this work we represented at the American Society for Human Genetics Annual Meeting (2013) and the Conference on Retroviruses and Opportunistic Infections (2014).

***Acknowledgements.*** We thank all contributing cohorts, and in particular the patients who accepted to participate in genetic studies.

The members of the SHCS are Aubert V, Battegay M, Bernasconi E, Böni J, Braun DL, Bucher HC, Calmy A, Cavassini M, Ciuffi A, Dollenmaier G, Egger M, Elzi L, Fehr J, Fellay J, Furrer H (Chairman of the Clinical and Laboratory Committee), Fux CA, Günthard HF (President of the SHCS), Haerry D (deputy of “Positive Council”), Hasse B, Hirsch HH, Hoffmann M, Hösli I, Kahlert C, Kaiser L, Keiser O, Klimkait T, Kouyos RD, Kovari H, Ledergerber B, Martinetti G, Martinez de Tejada B, Marzolini C, Metzner KJ, Müller N, Nicca D, Pantaleo G, Paioni P, Rauch A (Chairman of the Scientific Board), Rudin C (Chairman of the Mother and Child Substudy), Scherrer AU (Head of Data Centre), Schmid P, Speck R, Stöckle M, Tarr P, Trkola A, Vernazza P, Wandeler G, Weber R, Yerly S.

The members of the UK exome study are Richard Gilson (Mortimer Market Centre, London); Sabine Kinloch (Royal Free Hospital, London); Jane Deayton (Royal London Hospital, London); Clifford Lee (Western General Hospital, Edinburgh); Sarah Fidler (St Mary’s Hospital, London); Julie Fox (St Thomas’ Hospital, London); Christine Bowman (Royal Hallamshire Hospital, Sheffield); David Hawkins (Chelsea and Westminster Hospital, London); Harindra Veerakathy (St Mary’s Hospital, Portsmouth); Ken McLean (Charing Cross Hospital, London); Ashley Olson and Lorraine Fradette (University College, London); Claire Donnelly (Royal Victoria Hospital, Belfast); Mary Browning (Royal Infirmary, Cardiff); Johnathan Ross (Whittall Street Clinic, Birmingham); David Kellock (Kings Mill Centre, Sutton-in-Ashfield). Members of the CASCADE Collaboration in EuroCoord contributing data are the Amsterdam Cohort Studies among homosexual men and drug users, Amsterdam, Netherlands – Maria Prins; the Athens Multicentre AIDS Cohort Study (AMACS), Athens, Greece – Giota Touloumi; and St Pierre Hospital cohort, Brussels, Belgium – Stephan de Wit.

***Financial support.*** This study has been financed in part within the framework of the Swiss HIV Cohort Study (www.shcs.ch) project #651 and supported by the Swiss National Science Foundation (www.snf.ch) grant #148522 (J.F.). The International HIV Controllers Study was made possible through a generous donation from the Mark and Lisa Schwartz Foundation and a subsequent award from the Collaboration for AIDS Vaccine Discovery of the Bill and Melinda Gates Foundation (www.cavd.org). This work was also supported in part by the Harvard University Center for AIDS Research (cfar.globalhealth.harvard.edu) grant P-30-AI060354; University of California San Francisco (UCSF) Center for AIDS Research (cfar.ucsf.edu) grant P-30 AI27763; UCSF Clinical and Translational Science Institute (https://ctsi.ucsf.edu) grant UL1 RR024131; Center for AIDS Research Network of Integrated Clinical Systems (http://cfar.globalhealth.harvard.edu) grant R24 AI067039; and the National Institutes for Health (www.nih.gov) grants AI28568 and AI030914 (B.D.W.). The AIDS Clinical Trials Group was supported by NIH grants AI069513, AI34835, AI069432, AI069423, AI069477, AI069501, AI069474, AI069428, AI69467, AI069415, Al32782, AI27661, AI25859, AI28568, AI30914, AI069495, AI069471, AI069532, AI069452, AI069450, AI069556, AI069484, AI069472, AI34853, AI069465, AI069511, AI38844, AI069424, AI069434, AI46370, AI68634, AI069502, AI069419, AI068636, RR024975, AI077505, AI110527, and TR000445 (D.W.H.). For the CASCADE Consortium, the research leading to these results has received funding from the European Union Seventh Programme (FP7/2007–2013) under EuroCoord (www.eurocoord.net) grant agreement no. 260694 (K.P.) and the Spanish Network of HIV/AIDS grant nos. RD06/006, RD12/0017/0018 and RD16CIII/0002/0006 (J.DA.).

A portion of the data in this manuscript were collected by the Multicenter AIDS Cohort Study (MACS). MACS (Principal Investigators): Johns Hopkins University Bloomberg School of Public Health (Joseph Margolick, Todd Brown), U01-AI35042; Northwestern University (Steven Wolinsky), U01-AI35039; University of California, Los Angeles (Roger Detels, Oto Martinez-Maza, Otto Yang), U01-AI35040; University of Pittsburgh (Charles Rinaldo, Lawrence A. Kingsley, Jeremy J. Martinson), U01-AI35041; the Center for Analysis and Management of MACS, Johns Hopkins University Bloomberg School of Public Health (Lisa Jacobson, Gypsyamber D’Souza), UM1-AI35043. The MACS is funded primarily by the National Institute of Allergy and Infectious Diseases (NIAID), with additional co-funding from the National Cancer Institute (NCI), the National Institute on Drug Abuse (NIDA), and the National Institute of Mental Health (NIMH). Targeted supplemental funding for specific projects was also provided by the National Heart, Lung, and Blood Institute (NHLBI), and the National Institute on Deafness and Communication Disorders (NIDCD). MACS data collection is also supported by UL1-TR001079 (JHU ICTR) from the National Center for Advancing Translational Sciences (NCATS) a component of the National Institutes of Health (NIH), and NIH Roadmap for Medical Research. The contents of this publication are solely the responsibility of the authors and do not represent the official views of the National Institutes of Health (NIH), Johns Hopkins ICTR, or NCATS. The MACS website is located at http://aidscohortstudy.org/
