# Erratum

**DOI:** 10.1093/infdis/jiy226

**Published:** 2018-05-24

In “Evaluating the Impact of Functional Genetic Variation on HIV-1 Control” by McLaren et al. [J Infect Dis. 2017; 216: 1063–1069] the authors report that in the author order Steven M Wolinsky should be listed as number seventeen with the affiliation “Division of Infectious Diseases, The Feinberg School of Medicine, Northwestern University, Chicago”. Furthermore the authors would like to include the following in the acknowledgements: “A portion of the data in this manuscript were collected by the Multicenter AIDS Cohort Study (MACS). MACS (Principal Investigators): Johns Hopkins University Bloomberg School of Public Health (Joseph Margolick, Todd Brown), U01-AI35042; Northwestern University (Steven Wolinsky), U01-AI35039; University of California, Los Angeles (Roger Detels, Oto Martinez-Maza, Otto Yang), U01-AI35040; University of Pittsburgh (Charles Rinaldo, Lawrence A. Kingsley, Jeremy J. Martinson), U01-AI35041; the Center for Analysis and Management of MACS, Johns Hopkins University Bloomberg School of Public Health (Lisa Jacobson, Gypsyamber D’Souza), UM1-AI35043. The MACS is funded primarily by the National Institute of Allergy and Infectious Diseases (NIAID), with additional co-funding from the National Cancer Institute (NCI), the National Institute on Drug Abuse (NIDA), and the National Institute of Mental Health (NIMH). Targeted supplemental funding for specific projects was also provided by the National Heart, Lung, and Blood Institute (NHLBI), and the National Institute on Deafness and Communication Disorders (NIDCD). MACS data collection is also supported by UL1-TR001079 (JHU ICTR) from the National Center for Advancing Translational Sciences (NCATS) a component of the National Institutes of Health (NIH), and NIH Roadmap for Medical Research. The contents of this publication are solely the responsibility of the authors and do not represent the official views of the National Institutes of Health (NIH), Johns Hopkins ICTR, or NCATS. . The MACS website is located at http://aidscohortstudy.org/”

The authors regret the error.

