# Supplementary material for: Evaluating the Impact of Functional Genetic Variation on HIV-1 Control
Source: J Infect Dis. 2017 Sep 9;216(9):1063–9. doi: 10.1093/infdis/jix470 (PMC5853944; doi:10.1093/infdis/jix470)
Supplement: Supplementary Figure S1 [file jix470_suppl_supplementary_figure_s1.docx]

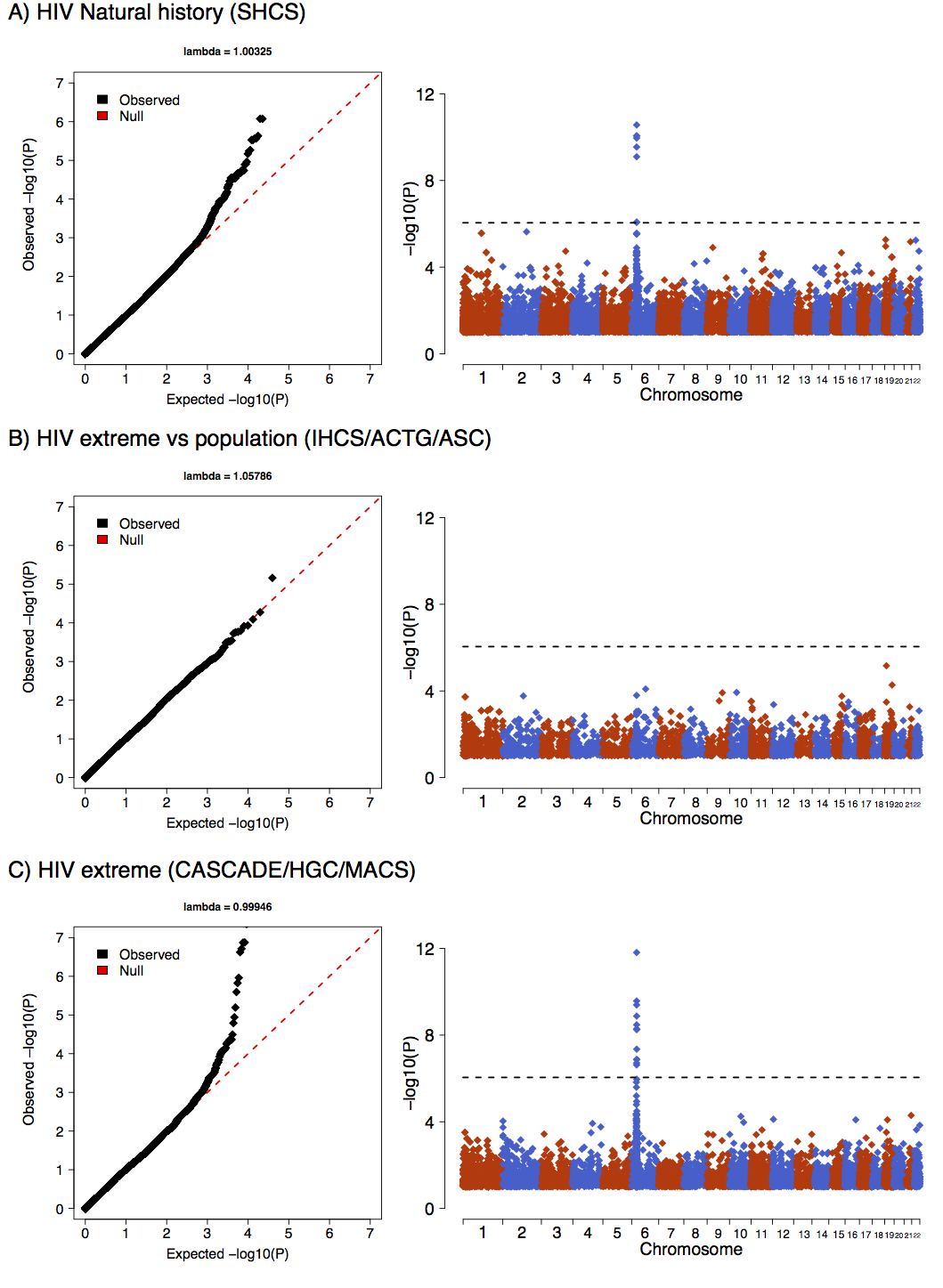


**Figure S1:** Single variant association results per phenotype group. Association results are shown per group for A) natural history of HIV progression B) comparison of HIV controllers vs HIV non-controllers/HIV- and C) comparison of extreme HIV progression phenotypes as defined in **Table 1** and **Table S1**. Quantile-quantile plots (left column) show the observed –log10 transformed p-value (y-axis) vs the null expectation (x-axis). All three groups show good control of genomic inflation (lambda~1). Manhattan plots (right column) show –log10 association p-values (y-axis) by genomic position (x-axis). No significant associations are observed outside of the MHC region on chromosome 6. For the study comparing HIV controllers vs HIV non-controllers/HIV- (panel B), HIV controllers without known protective HLA-B alleles were preferentially included resulting in a loss of MHC association signal in that group
