# Supplementary material for: Evaluating the Impact of Functional Genetic Variation on HIV-1 Control
Source: J Infect Dis. 2017 Sep 9;216(9):1063–9. doi: 10.1093/infdis/jix470 (PMC5853944; doi:10.1093/infdis/jix470)
Supplement: Supplementary Figure S2 [file jix470_suppl_supplementary_figure_s2.docx]

**Figure S2:** Power calculations for the SKAT burden test. We performed power tests using the power function built into the SKAT library in R. This function uses a reference dataset of 10,000 haplotypes to simulate burden testing power. We simulated a number of scenarios, varying the percent of causal (risk) and protective variation in the gene or gene pathway being tested. Power is shown for three significance (alpha) thresholds for each scenario
