# Supplementary material for: Evaluating the Impact of Functional Genetic Variation on HIV-1 Control
Source: J Infect Dis. 2017 Sep 9;216(9):1063–9. doi: 10.1093/infdis/jix470 (PMC5853944; doi:10.1093/infdis/jix470)
Supplement: Supplementary Table S1 [file jix470_suppl_supplementary_table_s1.docx]

**Table S1: Summary of samples, cohorts and phenotype definitions**

| **Analysis Group** | **Cohort Name** | **Abbreviation** | **N** | **European Ancestry (%)** | **Trait(s)** | **Definition(s)** |
| --- | --- | --- | --- | --- | --- | --- |
| Quantitative set point viral load | Swiss HIV Cohort Study | SHCS | 392 | 100% | Set point viral load (log10 RNA copies/ml of plasma) | Three or more stable plasma HIV RNA results in the absence of antiretroviral treatment, and meeting one of the following criteria: a valid seroconversion date estimation proven by documents or biological markers; or, for seroprevalent patients, VL data over a period of at least 3 years, diverging by no more than 0.5 log |
| HIV elite controllers and population controls | International HIV Controllers Study | IHCS | 219 | 100% | HIV elite control | Three or more measurements of plasma virus load (VL) < 50 RNA copies/ml over at least a 12-month period in the absence of antiviral therapy |
|  | AIDS Clinical Trials Group | ACTG | 64 | 100% | HIV non-controllers | Treatment-naïve, chronically HIV infected individuals enrolled in a study arm of the AIDS Clinical Trials Group |
|  | Autism Sequencing Consortium | ASC | 372 | 100% | HIV-negative | HIV negative indviduals used as controls by the Autism Sequencing Consortium |
| HIV controllers and rapid progressors | Multicenter AIDS Cohort Study | MACS | 88 | 100% | HiV-C/HIV-RP | HIV-C: Stable, ART-naïve viral load < 2,000 copies/ml and lack of progression to AIDS for 10 years  HIV-RP: Progression to CD4 counts <350/ul within 3 years of known or estimated seroconversion |
|  | HIV Genomics Consortium | HGC | 57 | 75% | HiV-C/HIV-RP | Phase1 (n=45) HIV-C: ART naïve HIV infection for >10 years with all CD4 counts > 500/ul and at least three viral RNA measurements <50 copies/ml with the first and last measurement at least 12 months apart  HIV-RP: HIV-1 RNA levels > 100,000 copies/ml within three years of the estimated seroconversion date and either one of the following: 1. At least two consecutive CD4 lymphocyte counts < 200/ul within three years of seroconversion or 2. At least one CD4 count <200/ul and a diagnosis of AIDS within three years of seroconversion.  Phase 2 (n=12) HIV-C: ART naïve, HIV+ for ≥ 1 year with ≥ 3 consecutive viral loads <75 copies/ml over one year with all previous viral loads <1000 copies/ml OR HIV+ for ≥ 10 years, with ≥ 2 viral loads and ≥ 90% of all viral loads <400 copies/ml  HIV-RP: HIV positive individual with ≥1 CD4 count ≤200 cells/mm3 within 6 months of estimated seroconversion |
|  | CASCADE | CASCADE | 183 | 75% | HiV-C/HIV-RP | HIV-C: ART naïve, HIV+ for ≥ 1 year with ≥ 3 consecutive viral loads <75 copies/ml over one year with all previous viral loads <1000 copies/mlORHIV+ for ≥ 10 years, with ≥ 2 viral loads and ≥ 90% of all viral loads <400 copies/mlHIV-RP: HIV positive individual with ≥1 CD4 count ≤200 cells/mm3 within 6 months of estimated seroconversion |
