# Supplementary material for: Evaluating the Impact of Functional Genetic Variation on HIV-1 Control
Source: J Infect Dis. 2017 Sep 9;216(9):1063–9. doi: 10.1093/infdis/jix470 (PMC5853944; doi:10.1093/infdis/jix470)
Supplement: Supplementary Table S2 [file jix470_suppl_supplementary_table_s2.docx]

**Table S2: Summary of sequencing performance per analysis group**

| **Analysis Group** | **Cohort Name** | **Abbreviation** | **N** | **Capture kit** | **TiTv  Mean (sd)** | **N SNPs  Mean (sd)** | **N Indels  Mean (sd)** |
| --- | --- | --- | --- | --- | --- | --- | --- |
| Quantitative set point viral load | Swiss HIV Cohort Study | SHCS | 392 | Illumina Truseq 65Mb | 2.54 (0.02) | 45277 (610) | 7837 (310) |
| HIV elite controllers and HIV negative population controls | International HIV Controllers Study | IHCS | 219 | Agilent 38Mb SureSelect v2 | 2.92 (0.04) | 22505 (1100) | NA |
|  | AIDS Clinical Trials Group | ACTG | 64 | Agilent 38Mb SureSelect v2 | 2.90 (0.04) | 23993 (984) | NA |
|  | Autism Sequencing Consortium | ASC | 372 | Agilent 38Mb SureSelect v2 | 2.92 (0.08) | 23981 (2274) | NA |
| HIV controllers and rapid progressors | Multicenter AIDS Cohort Study | MACS | 88 | Agilent SureSelect Human All Exon 37Mb v1 | 2.86 (0.04) | 24405 (2205) | 1631 (266) |
|  | HIV Genomics Consortium | HGC | 57 | Agilent SureSelect Human All Exon 50Mb v5 | 2.82 (0.08) | 26450 (3856) | 1570 (918) |
|  | CASCADE | CASCADE | 183 | Agilent SureSelect Human All Exon 50Mb v5 | 2.71 (0.03) | 29004 (2274) | 1916 (155) |
