# Supplementary material for: Evaluating the Impact of Functional Genetic Variation on HIV-1 Control
Source: J Infect Dis. 2017 Sep 9;216(9):1063–9. doi: 10.1093/infdis/jix470 (PMC5853944; doi:10.1093/infdis/jix470)
Supplement: Supplementary Table S3 [file jix470_suppl_supplementary_table_s3.docx]

**Table S3: Gene association results for HIV Dependency Factors**

| **Gene Symbol** | **CRISPR^a^** | **siRNA FDR 0.2^a^** | **siRNA FDR 0.05^a^** | **MED^a^** | **NPC^a^** | **P All Variants** | **P Protein Changing^b^** | **P High Impact^b^** |
| --- | --- | --- | --- | --- | --- | --- | --- | --- |
| MMADHC | 0 | 1 | 0 | 0 | 0 | 4.00E-05 | 3.98E-05 | NA |
| C9orf131 | 0 | 1 | 1 | 0 | 0 | 2.29E-03 | 2.79E-03 | 2.14E-01 |
| PPP1R18 | 0 | 1 | 0 | 0 | 0 | 5.04E-03 | 5.04E-03 | NA |
| RHOB | 0 | 1 | 0 | 0 | 0 | 6.36E-03 | NA | NA |
| UMPS | 0 | 1 | 0 | 0 | 0 | 8.09E-03 | 1.83E-02 | NA |
| PAX2 | 0 | 1 | 0 | 0 | 0 | 9.70E-03 | 8.66E-01 | NA |
| U2SURP | 0 | 1 | 0 | 0 | 0 | 1.10E-02 | 5.81E-01 | NA |
| SLC7A6OS | 0 | 1 | 0 | 0 | 0 | 1.42E-02 | 3.69E-03 | NA |
| FOXP2 | 0 | 1 | 0 | 0 | 0 | 1.59E-02 | 2.86E-02 | NA |
| GLRA4 | 0 | 1 | 0 | 0 | 0 | 1.79E-02 | 3.43E-02 | 1.53E-02 |
| C11orf87 | 0 | 1 | 0 | 0 | 0 | 1.80E-02 | 3.98E-01 | NA |
| SEC13 | 0 | 1 | 1 | 0 | 1 | 1.85E-02 | 1.69E-01 | NA |
| ARHGAP27 | 0 | 1 | 0 | 0 | 0 | 1.89E-02 | 2.73E-02 | 5.50E-01 |
| EFCAB2 | 0 | 1 | 1 | 0 | 0 | 2.05E-02 | 2.25E-02 | NA |
| RPAP3 | 0 | 1 | 0 | 0 | 0 | 2.06E-02 | 6.89E-03 | NA |
| PARVB | 0 | 1 | 0 | 0 | 0 | 2.52E-02 | 1.10E-01 | NA |
| MYL1 | 0 | 1 | 0 | 0 | 0 | 2.69E-02 | 2.93E-02 | 1.53E-01 |
| CCR5 | 1 | 0 | 0 | 0 | 0 | 2.79E-02 | 4.53E-02 | 3.07E-01 |
| TMEM128 | 0 | 1 | 0 | 0 | 0 | 2.80E-02 | 2.90E-02 | NA |
| MTX2 | 0 | 1 | 0 | 0 | 0 | 2.98E-02 | 7.40E-02 | NA |
| COPB2 | 0 | 1 | 1 | 0 | 0 | 3.01E-02 | 1.05E-01 | NA |
| ETF1 | 0 | 1 | 0 | 0 | 0 | 3.02E-02 | NA | NA |
| C4orf27 | 0 | 1 | 0 | 0 | 0 | 3.25E-02 | 2.62E-02 | 9.69E-01 |
| CTR9 | 0 | 1 | 0 | 0 | 0 | 3.29E-02 | 1.24E-01 | NA |
| BTRC | 0 | 1 | 0 | 0 | 0 | 3.35E-02 | 2.85E-02 | 5.16E-02 |
| ZNF720 | 0 | 1 | 0 | 0 | 0 | 3.39E-02 | 3.53E-01 | NA |
| HDAC7 | 0 | 1 | 0 | 0 | 0 | 3.50E-02 | 1.61E-01 | NA |
| E4F1 | 0 | 1 | 1 | 0 | 0 | 3.70E-02 | 2.60E-02 | NA |
| PAIP1 | 0 | 1 | 1 | 0 | 0 | 3.77E-02 | 3.97E-01 | 3.97E-01 |
| EXT1 | 0 | 1 | 1 | 0 | 0 | 3.91E-02 | 4.79E-02 | NA |
| CDC40 | 0 | 1 | 0 | 0 | 0 | 3.92E-02 | 1.66E-02 | NA |
| ACTG1 | 0 | 1 | 0 | 0 | 0 | 4.12E-02 | 6.20E-02 | NA |
| NUP160 | 0 | 1 | 1 | 0 | 1 | 4.64E-02 | 4.96E-01 | NA |
| UPF3B | 0 | 1 | 0 | 0 | 0 | 4.70E-02 | 1.15E-01 | NA |
| MED11 | 0 | 1 | 1 | 1 | 0 | 4.89E-02 | 4.89E-02 | 1.08E-02 |
| HPGD | 0 | 1 | 1 | 0 | 0 | 4.97E-02 | 2.39E-02 | NA |
| HIP1R | 0 | 1 | 0 | 0 | 0 | 4.98E-02 | 3.14E-02 | NA |
| MED16 | 0 | 1 | 0 | 1 | 0 | 4.98E-02 | 1.62E-01 | 7.61E-01 |
| MYH10 | 0 | 1 | 0 | 0 | 0 | 5.11E-02 | 2.15E-02 | NA |
| KLRF1 | 0 | 1 | 0 | 0 | 0 | 5.30E-02 | 5.55E-02 | NA |
| OR11H6 | 0 | 1 | 0 | 0 | 0 | 5.45E-02 | 5.28E-02 | 7.31E-01 |
| LAMTOR5 | 0 | 1 | 0 | 0 | 0 | 5.83E-02 | 3.79E-01 | NA |
| CCDC121 | 0 | 1 | 1 | 0 | 0 | 5.85E-02 | 1.45E-01 | 9.57E-01 |
| CLUAP1 | 0 | 1 | 0 | 0 | 0 | 5.85E-02 | 1.65E-01 | NA |
| SPAG1 | 0 | 1 | 0 | 0 | 0 | 6.16E-02 | 8.83E-01 | NA |
| CARD11 | 0 | 1 | 0 | 0 | 0 | 6.16E-02 | 2.73E-02 | NA |
| CCND1 | 0 | 1 | 0 | 0 | 0 | 6.24E-02 | NA | NA |
| COG2 | 0 | 1 | 0 | 0 | 0 | 6.35E-02 | 8.06E-02 | 8.43E-01 |
| PRPS1 | 0 | 1 | 0 | 0 | 0 | 6.48E-02 | NA | NA |
| RFPL4A | 0 | 1 | 0 | 0 | 0 | 6.64E-02 | 1.37E-02 | NA |
| NUP62 | 0 | 1 | 0 | 0 | 1 | 6.78E-02 | 1.07E-02 | NA |
| APOBEC3G | 0 | 1 | 0 | 0 | 0 | 7.29E-02 | 4.84E-02 | 5.65E-02 |
| NUP54 | 0 | 1 | 0 | 0 | 1 | 7.47E-02 | 4.13E-01 | NA |
| SRP14 | 0 | 1 | 0 | 0 | 0 | 7.48E-02 | 6.21E-02 | NA |
| EXOC3L4 | 0 | 1 | 0 | 0 | 0 | 7.86E-02 | 4.85E-02 | NA |
| GRHL3 | 0 | 1 | 0 | 0 | 0 | 7.92E-02 | 1.93E-01 | NA |
| STK39 | 0 | 1 | 0 | 0 | 0 | 8.22E-02 | 3.79E-02 | NA |
| ASPA | 0 | 1 | 0 | 0 | 0 | 8.25E-02 | 1.96E-01 | NA |
| CRNKL1 | 0 | 1 | 1 | 0 | 0 | 8.36E-02 | 8.38E-02 | 3.21E-01 |
| C17orf50 | 0 | 1 | 0 | 0 | 0 | 8.46E-02 | 8.33E-02 | NA |
| SLC22A14 | 0 | 1 | 0 | 0 | 0 | 8.67E-02 | 5.40E-02 | 1.34E-01 |
| RAD9B | 0 | 1 | 0 | 0 | 0 | 8.73E-02 | 6.96E-02 | NA |
| E2F5 | 0 | 1 | 0 | 0 | 0 | 8.86E-02 | 8.83E-02 | NA |
| VSTM2L | 0 | 1 | 0 | 0 | 0 | 8.95E-02 | 1.49E-01 | NA |
| CENPM | 0 | 1 | 0 | 0 | 0 | 9.12E-02 | 2.61E-01 | NA |
| AHSP | 0 | 1 | 0 | 0 | 0 | 9.15E-02 | 1.03E-01 | NA |
| ANKRD28 | 0 | 1 | 0 | 0 | 0 | 9.28E-02 | 1.36E-01 | NA |
| NUPL2 | 0 | 1 | 0 | 0 | 1 | 9.29E-02 | 3.64E-01 | 8.98E-02 |
| NUP35 | 0 | 1 | 0 | 0 | 1 | 9.42E-02 | 1.50E-01 | NA |
| EP300 | 0 | 1 | 0 | 0 | 0 | 9.48E-02 | 4.81E-01 | NA |
| RAB33B | 0 | 1 | 1 | 0 | 0 | 9.59E-02 | 9.58E-02 | NA |
| NFE2L1 | 0 | 1 | 0 | 0 | 0 | 9.82E-02 | 8.21E-01 | NA |
| NUP153 | 0 | 1 | 1 | 0 | 1 | 1.01E-01 | 3.28E-01 | NA |
| SNRPA1 | 0 | 1 | 0 | 0 | 0 | 1.02E-01 | 1.40E-01 | NA |
| SLC38A6 | 0 | 1 | 0 | 0 | 0 | 1.03E-01 | 9.56E-02 | 9.04E-02 |
| RGP1 | 0 | 1 | 0 | 0 | 0 | 1.05E-01 | 1.05E-01 | NA |
| CCDC158 | 0 | 1 | 0 | 0 | 0 | 1.06E-01 | 7.55E-02 | NA |
| SF3A1 | 0 | 1 | 0 | 0 | 0 | 1.06E-01 | 1.53E-02 | NA |
| CXorf21 | 0 | 1 | 0 | 0 | 0 | 1.10E-01 | 1.10E-01 | NA |
| RGPD3 | 0 | 1 | 0 | 0 | 0 | 1.19E-01 | 9.95E-02 | 9.04E-01 |
| TMED8 | 0 | 1 | 0 | 0 | 0 | 1.20E-01 | 3.08E-01 | NA |
| FAM57A | 0 | 1 | 0 | 0 | 0 | 1.21E-01 | 3.67E-01 | NA |
| NUPL1 | 0 | 1 | 0 | 0 | 1 | 1.22E-01 | 1.40E-01 | NA |
| ADPRHL1 | 0 | 1 | 1 | 0 | 0 | 1.23E-01 | 2.70E-01 | 8.40E-01 |
| TLR1 | 0 | 1 | 0 | 0 | 0 | 1.26E-01 | 1.12E-01 | 8.30E-01 |
| ITGA4 | 0 | 1 | 0 | 0 | 0 | 1.27E-01 | 2.94E-01 | NA |
| EGLN3 | 0 | 1 | 0 | 0 | 0 | 1.31E-01 | 7.17E-01 | 3.13E-01 |
| TPR | 0 | 1 | 0 | 0 | 1 | 1.32E-01 | 2.52E-02 | NA |
| LOH12CR2 | 0 | 1 | 0 | 0 | 0 | 1.32E-01 | 1.32E-01 | NA |
| POLR2J | 0 | 1 | 1 | 0 | 0 | 1.33E-01 | NA | NA |
| HES1 | 0 | 1 | 0 | 0 | 0 | 1.35E-01 | 2.82E-01 | NA |
| MED21 | 0 | 1 | 1 | 1 | 0 | 1.41E-01 | 1.41E-01 | NA |
| RPL28 | 0 | 1 | 0 | 0 | 0 | 1.42E-01 | 8.31E-02 | NA |
| MZT1 | 0 | 1 | 0 | 0 | 0 | 1.45E-01 | 2.48E-01 | NA |
| KRT40 | 0 | 1 | 0 | 0 | 0 | 1.51E-01 | 3.37E-01 | 1.54E-01 |
| VPS9D1 | 0 | 1 | 1 | 0 | 0 | 1.52E-01 | 5.88E-01 | NA |
| TNPO3 | 0 | 1 | 1 | 0 | 0 | 1.53E-01 | 1.08E-02 | NA |
| NT5C3L | 0 | 1 | 0 | 0 | 0 | 1.57E-01 | 1.64E-01 | NA |
| HSPD1 | 0 | 1 | 0 | 0 | 0 | 1.58E-01 | 8.14E-02 | NA |
| ZNF367 | 0 | 1 | 0 | 0 | 0 | 1.59E-01 | 4.58E-01 | NA |
| SLC2A14 | 0 | 1 | 0 | 0 | 0 | 1.59E-01 | 7.63E-01 | NA |
| DSG4 | 0 | 1 | 0 | 0 | 0 | 1.62E-01 | 6.74E-02 | 3.72E-01 |
| EIF4A3 | 0 | 1 | 1 | 0 | 0 | 1.63E-01 | NA | NA |
| HDGFL1 | 0 | 1 | 0 | 0 | 0 | 1.66E-01 | 1.54E-01 | NA |
| HDAC2 | 0 | 1 | 0 | 0 | 0 | 1.66E-01 | 4.22E-01 | NA |
| PSMD1 | 0 | 1 | 1 | 0 | 0 | 1.67E-01 | 3.83E-01 | NA |
| MED19 | 0 | 1 | 0 | 1 | 0 | 1.67E-01 | 7.35E-01 | NA |
| KIN | 0 | 1 | 0 | 0 | 0 | 1.67E-01 | 6.91E-01 | 6.91E-01 |
| SYT8 | 0 | 1 | 0 | 0 | 0 | 1.68E-01 | 8.95E-02 | 7.18E-01 |
| MED6 | 0 | 1 | 1 | 1 | 0 | 1.68E-01 | 2.91E-01 | NA |
| OR1K1 | 0 | 1 | 0 | 0 | 0 | 1.70E-01 | 1.70E-01 | NA |
| OR2F1 | 0 | 1 | 0 | 0 | 0 | 1.71E-01 | 3.40E-01 | 7.28E-01 |
| FAM154B | 0 | 1 | 1 | 0 | 0 | 1.74E-01 | 1.35E-01 | 4.89E-01 |
| LARP6 | 0 | 1 | 0 | 0 | 0 | 1.75E-01 | 6.43E-01 | NA |
| WBP2 | 0 | 1 | 0 | 0 | 0 | 1.80E-01 | 8.55E-01 | NA |
| GINS4 | 0 | 1 | 0 | 0 | 0 | 1.83E-01 | 7.87E-01 | NA |
| HNRNPR | 0 | 1 | 0 | 0 | 0 | 1.86E-01 | 6.99E-01 | NA |
| CCL8 | 0 | 1 | 0 | 0 | 0 | 1.87E-01 | 2.71E-01 | NA |
| PSMA3 | 0 | 1 | 0 | 0 | 0 | 1.87E-01 | 7.76E-02 | NA |
| XAB2 | 0 | 1 | 1 | 0 | 0 | 1.88E-01 | 3.76E-01 | NA |
| GAL | 0 | 1 | 0 | 0 | 0 | 1.90E-01 | 1.54E-01 | NA |
| SUPV3L1 | 0 | 1 | 0 | 0 | 0 | 1.90E-01 | 2.19E-01 | NA |
| PSMB6 | 0 | 1 | 0 | 0 | 0 | 1.96E-01 | 1.68E-01 | NA |
| NDUFA6 | 0 | 1 | 0 | 0 | 0 | 1.96E-01 | 3.22E-01 | NA |
| CLDND1 | 0 | 1 | 0 | 0 | 0 | 1.98E-01 | 3.97E-01 | NA |
| C3orf17 | 0 | 1 | 0 | 0 | 0 | 1.99E-01 | 1.92E-01 | NA |
| ANKRD30A | 0 | 1 | 0 | 0 | 0 | 2.01E-01 | 1.64E-01 | 6.36E-01 |
| BTN3A1 | 0 | 1 | 0 | 0 | 0 | 2.09E-01 | 8.13E-02 | 2.40E-01 |
| MAGIX | 0 | 1 | 0 | 0 | 0 | 2.12E-01 | 3.61E-01 | NA |
| PCDHA3 | 0 | 1 | 0 | 0 | 0 | 2.13E-01 | 5.54E-01 | 4.35E-01 |
| CST8 | 0 | 1 | 0 | 0 | 0 | 2.16E-01 | 4.80E-01 | NA |
| POP1 | 0 | 1 | 0 | 0 | 0 | 2.16E-01 | 2.42E-01 | 3.05E-01 |
| TBC1D28 | 0 | 1 | 1 | 0 | 0 | 2.18E-01 | 2.51E-01 | NA |
| C3orf58 | 0 | 1 | 0 | 0 | 0 | 2.22E-01 | 4.45E-01 | NA |
| NUP50 | 0 | 1 | 0 | 0 | 1 | 2.24E-01 | 8.88E-01 | NA |
| RBMX2 | 0 | 1 | 0 | 0 | 0 | 2.30E-01 | 3.86E-01 | NA |
| ASB9 | 0 | 1 | 0 | 0 | 0 | 2.30E-01 | 2.30E-01 | NA |
| GPR21 | 0 | 1 | 0 | 0 | 0 | 2.32E-01 | 1.95E-01 | NA |
| CD163L1 | 0 | 1 | 0 | 0 | 0 | 2.37E-01 | 5.81E-01 | 2.41E-01 |
| GABRG3 | 0 | 1 | 0 | 0 | 0 | 2.38E-01 | 2.46E-01 | NA |
| PGGT1B | 0 | 1 | 0 | 0 | 0 | 2.38E-01 | 2.93E-01 | NA |
| SCUBE1 | 0 | 1 | 0 | 0 | 0 | 2.41E-01 | 3.42E-01 | 3.78E-01 |
| FBXO18 | 0 | 1 | 0 | 0 | 0 | 2.45E-01 | 2.50E-01 | NA |
| UEVLD | 0 | 1 | 0 | 0 | 0 | 2.45E-01 | 3.65E-01 | NA |
| ARSK | 0 | 1 | 0 | 0 | 0 | 2.53E-01 | 7.17E-01 | NA |
| LRRC47 | 0 | 1 | 0 | 0 | 0 | 2.53E-01 | 8.05E-01 | NA |
| KERA | 0 | 1 | 0 | 0 | 0 | 2.53E-01 | 7.34E-01 | 6.01E-01 |
| RIMS2 | 0 | 1 | 0 | 0 | 0 | 2.54E-01 | 1.77E-02 | NA |
| ETFB | 0 | 1 | 0 | 0 | 0 | 2.58E-01 | 4.99E-01 | NA |
| MED22 | 0 | 1 | 0 | 1 | 0 | 2.62E-01 | 8.51E-01 | 7.72E-01 |
| KEAP1 | 0 | 1 | 0 | 0 | 0 | 2.63E-01 | 8.80E-01 | NA |
| SOGA2 | 0 | 1 | 0 | 0 | 0 | 2.66E-01 | 1.13E-01 | 2.03E-01 |
| PSMC4 | 0 | 1 | 0 | 0 | 0 | 2.70E-01 | 6.28E-01 | NA |
| SCFD1 | 0 | 1 | 1 | 0 | 0 | 2.74E-01 | 2.56E-01 | 3.82E-01 |
| C4orf33 | 0 | 1 | 0 | 0 | 0 | 2.78E-01 | 7.45E-01 | 6.22E-01 |
| BUD31 | 0 | 1 | 0 | 0 | 0 | 2.79E-01 | 2.70E-01 | NA |
| MED20 | 0 | 1 | 0 | 1 | 0 | 2.80E-01 | NA | NA |
| LETM1 | 0 | 1 | 0 | 0 | 0 | 2.82E-01 | 1.15E-01 | NA |
| MED1 | 0 | 1 | 0 | 1 | 0 | 2.83E-01 | 6.21E-01 | NA |
| DHRS7C | 0 | 1 | 0 | 0 | 0 | 2.84E-01 | 3.10E-02 | 8.13E-01 |
| MED4 | 0 | 1 | 1 | 1 | 0 | 2.90E-01 | 3.65E-01 | NA |
| PRPF6 | 0 | 1 | 0 | 0 | 0 | 2.91E-01 | 2.01E-01 | 1.80E-01 |
| GLRX5 | 0 | 1 | 0 | 0 | 0 | 3.00E-01 | 7.01E-01 | NA |
| PRR19 | 0 | 1 | 0 | 0 | 0 | 3.01E-01 | 2.86E-01 | NA |
| HMBS | 0 | 1 | 0 | 0 | 0 | 3.01E-01 | 2.51E-01 | NA |
| MED17 | 0 | 1 | 0 | 1 | 0 | 3.01E-01 | 5.98E-01 | NA |
| GSX2 | 0 | 1 | 0 | 0 | 0 | 3.04E-01 | 7.86E-01 | NA |
| ARAP1 | 0 | 1 | 0 | 0 | 0 | 3.08E-01 | 8.29E-01 | 7.38E-01 |
| LILRA1 | 0 | 1 | 0 | 0 | 0 | 3.08E-01 | 2.89E-01 | NA |
| RAB19 | 0 | 1 | 0 | 0 | 0 | 3.17E-01 | 9.95E-01 | NA |
| PNISR | 0 | 1 | 0 | 0 | 0 | 3.23E-01 | 8.78E-02 | NA |
| ZC3H7B | 0 | 1 | 0 | 0 | 0 | 3.24E-01 | 5.02E-01 | NA |
| RGS9BP | 0 | 1 | 0 | 0 | 0 | 3.35E-01 | 5.39E-01 | 4.15E-01 |
| FAM86DP | 0 | 1 | 0 | 0 | 0 | 3.39E-01 | NA | NA |
| TPST2 | 1 | 0 | 0 | 0 | 0 | 3.39E-01 | 4.50E-02 | NA |
| HYI | 0 | 1 | 0 | 0 | 0 | 3.41E-01 | NA | NA |
| PHF2 | 0 | 1 | 1 | 0 | 0 | 3.44E-01 | 7.74E-02 | NA |
| CXCR4 | 0 | 1 | 1 | 0 | 0 | 3.45E-01 | 1.88E-01 | NA |
| DTNBP1 | 0 | 1 | 0 | 0 | 0 | 3.46E-01 | 8.58E-01 | NA |
| WNT10A | 0 | 1 | 0 | 0 | 0 | 3.47E-01 | 3.11E-01 | NA |
| SYNCRIP | 0 | 1 | 0 | 0 | 0 | 3.48E-01 | 3.48E-01 | NA |
| EEF1A1 | 0 | 1 | 0 | 0 | 0 | 3.48E-01 | 4.72E-01 | NA |
| WHSC1 | 0 | 1 | 0 | 0 | 0 | 3.48E-01 | 1.01E-01 | NA |
| OTUD1 | 0 | 1 | 0 | 0 | 0 | 3.51E-01 | 5.05E-01 | NA |
| LRRC8E | 0 | 1 | 0 | 0 | 0 | 3.53E-01 | 2.56E-01 | 8.19E-01 |
| ATAT1 | 0 | 1 | 0 | 0 | 0 | 3.56E-01 | 1.53E-01 | NA |
| GNB2 | 0 | 1 | 0 | 0 | 0 | 3.60E-01 | 3.74E-01 | NA |
| LCE1C | 0 | 1 | 1 | 0 | 0 | 3.63E-01 | 3.78E-01 | NA |
| C20orf85 | 0 | 1 | 0 | 0 | 0 | 3.64E-01 | 1.59E-01 | NA |
| RICTOR | 0 | 1 | 0 | 0 | 0 | 3.66E-01 | 1.14E-01 | NA |
| RNF38 | 0 | 1 | 0 | 0 | 0 | 3.67E-01 | 5.17E-01 | 2.15E-01 |
| DMPK | 0 | 1 | 0 | 0 | 0 | 3.68E-01 | 5.56E-02 | NA |
| SKIV2L2 | 0 | 1 | 0 | 0 | 0 | 3.69E-01 | 3.69E-01 | NA |
| SF3A2 | 0 | 1 | 1 | 0 | 0 | 3.70E-01 | 3.29E-01 | NA |
| ARIH2 | 0 | 1 | 0 | 0 | 0 | 3.71E-01 | 5.78E-01 | NA |
| ATIC | 0 | 1 | 1 | 0 | 0 | 3.76E-01 | 1.61E-01 | 7.00E-01 |
| RAP1B | 0 | 1 | 1 | 0 | 0 | 3.81E-01 | NA | NA |
| FBL | 0 | 1 | 0 | 0 | 0 | 3.82E-01 | 2.18E-02 | NA |
| CRB3 | 0 | 1 | 1 | 0 | 0 | 3.84E-01 | NA | NA |
| TTPA | 0 | 1 | 0 | 0 | 0 | 3.85E-01 | 2.34E-01 | NA |
| RCE1 | 0 | 1 | 0 | 0 | 0 | 3.87E-01 | 6.15E-01 | NA |
| ENPP3 | 0 | 1 | 0 | 0 | 0 | 3.91E-01 | 1.21E-01 | 3.51E-01 |
| HOMEZ | 0 | 1 | 0 | 0 | 0 | 3.92E-01 | 3.85E-01 | 8.31E-01 |
| GMEB2 | 0 | 1 | 0 | 0 | 0 | 3.93E-01 | 3.43E-01 | NA |
| HNRNPC | 0 | 1 | 1 | 0 | 0 | 3.96E-01 | 9.66E-02 | NA |
| IFFO2 | 0 | 1 | 1 | 0 | 0 | 3.97E-01 | 5.21E-01 | NA |
| SCARB1 | 0 | 1 | 1 | 0 | 0 | 3.97E-01 | 5.09E-01 | NA |
| GTF2E2 | 0 | 1 | 0 | 0 | 0 | 3.98E-01 | 3.98E-01 | NA |
| ZBTB43 | 0 | 1 | 0 | 0 | 0 | 3.98E-01 | 2.86E-01 | NA |
| MCM3AP | 0 | 1 | 0 | 0 | 0 | 3.99E-01 | 3.86E-02 | NA |
| SEC61B | 0 | 1 | 1 | 0 | 0 | 4.01E-01 | 4.77E-01 | NA |
| C17orf67 | 0 | 1 | 0 | 0 | 0 | 4.02E-01 | 3.73E-01 | 4.81E-01 |
| CHST15 | 0 | 1 | 0 | 0 | 0 | 4.06E-01 | 1.43E-01 | 7.22E-01 |
| ALCAM | 1 | 0 | 0 | 0 | 0 | 4.13E-01 | 9.45E-01 | NA |
| ACOT7 | 0 | 1 | 0 | 0 | 0 | 4.13E-01 | 4.92E-01 | NA |
| ACTC1 | 0 | 1 | 0 | 0 | 0 | 4.16E-01 | NA | NA |
| CHRNA10 | 0 | 1 | 0 | 0 | 0 | 4.19E-01 | 4.17E-01 | NA |
| MS4A8B | 0 | 1 | 0 | 0 | 0 | 4.19E-01 | 2.59E-01 | NA |
| C1orf194 | 0 | 1 | 0 | 0 | 0 | 4.24E-01 | 4.21E-01 | 4.76E-01 |
| TOM1L2 | 0 | 1 | 0 | 0 | 0 | 4.26E-01 | 5.42E-01 | 5.80E-01 |
| RELA | 0 | 1 | 1 | 0 | 0 | 4.27E-01 | 3.89E-01 | NA |
| B4GALNT4 | 0 | 1 | 0 | 0 | 0 | 4.29E-01 | 5.87E-01 | NA |
| SRSF2 | 0 | 1 | 1 | 0 | 0 | 4.30E-01 | 6.07E-01 | NA |
| FAM24A | 0 | 1 | 0 | 0 | 0 | 4.31E-01 | 2.66E-01 | NA |
| PTS | 0 | 1 | 0 | 0 | 0 | 4.32E-01 | 5.52E-01 | NA |
| CARM1 | 0 | 1 | 0 | 0 | 0 | 4.33E-01 | 5.58E-01 | NA |
| MED8 | 0 | 1 | 0 | 1 | 0 | 4.33E-01 | 5.12E-02 | NA |
| HAMP | 0 | 1 | 0 | 0 | 0 | 4.36E-01 | 4.06E-01 | NA |
| ARL16 | 0 | 1 | 0 | 0 | 0 | 4.37E-01 | 4.37E-01 | 4.37E-01 |
| SAMM50 | 0 | 1 | 0 | 0 | 0 | 4.40E-01 | 5.18E-01 | NA |
| OR2AT4 | 0 | 1 | 1 | 0 | 0 | 4.42E-01 | 8.94E-01 | NA |
| CYTH3 | 0 | 1 | 0 | 0 | 0 | 4.47E-01 | 8.82E-01 | NA |
| UHRF1 | 0 | 1 | 0 | 0 | 0 | 4.47E-01 | NA | NA |
| CTDP1 | 0 | 1 | 1 | 0 | 0 | 4.50E-01 | 1.22E-01 | NA |
| CHADL | 0 | 1 | 0 | 0 | 0 | 4.53E-01 | 8.06E-01 | 3.74E-01 |
| AKAP17A | 0 | 1 | 1 | 0 | 0 | 4.60E-01 | 8.00E-01 | NA |
| RPL12 | 0 | 1 | 0 | 0 | 0 | 4.61E-01 | 3.36E-01 | NA |
| UQCR10 | 0 | 1 | 1 | 0 | 0 | 4.65E-01 | 5.16E-01 | NA |
| NUP85 | 0 | 1 | 0 | 0 | 1 | 4.67E-01 | 6.87E-01 | NA |
| PHF17 | 0 | 1 | 0 | 0 | 0 | 4.68E-01 | 5.35E-01 | NA |
| ZNF688 | 0 | 1 | 0 | 0 | 0 | 4.73E-01 | 4.53E-01 | NA |
| SPP2 | 0 | 1 | 0 | 0 | 0 | 4.75E-01 | 2.76E-01 | 1.37E-01 |
| PSMA7 | 0 | 1 | 1 | 0 | 0 | 4.77E-01 | NA | NA |
| NUP188 | 0 | 1 | 0 | 0 | 1 | 4.83E-01 | 4.53E-01 | NA |
| CYCS | 0 | 1 | 0 | 0 | 0 | 4.84E-01 | 6.52E-01 | NA |
| POLR2I | 0 | 1 | 1 | 0 | 0 | 4.84E-01 | 8.95E-01 | 8.95E-01 |
| CCNT1 | 0 | 1 | 1 | 0 | 0 | 4.85E-01 | 4.60E-01 | 5.56E-01 |
| CDK19 | 0 | 1 | 0 | 0 | 0 | 4.86E-01 | NA | NA |
| MEGF10 | 0 | 1 | 0 | 0 | 0 | 4.87E-01 | 4.38E-01 | NA |
| IFT20 | 0 | 1 | 0 | 0 | 0 | 4.89E-01 | 5.28E-01 | NA |
| MGAT1 | 0 | 1 | 0 | 0 | 0 | 4.91E-01 | 2.70E-01 | NA |
| MED9 | 0 | 1 | 0 | 1 | 0 | 4.97E-01 | 6.56E-01 | NA |
| NUP37 | 0 | 1 | 0 | 0 | 1 | 4.98E-01 | 7.43E-01 | NA |
| POLR3F | 0 | 1 | 1 | 0 | 0 | 4.99E-01 | 2.43E-01 | 2.43E-01 |
| NPAS3 | 0 | 1 | 0 | 0 | 0 | 5.04E-01 | 2.86E-01 | NA |
| NCAPH2 | 0 | 1 | 0 | 0 | 0 | 5.06E-01 | 5.51E-01 | NA |
| THOC2 | 0 | 1 | 1 | 0 | 0 | 5.06E-01 | 5.39E-01 | NA |
| CRTC2 | 0 | 1 | 0 | 0 | 0 | 5.08E-01 | 6.16E-01 | NA |
| TDRD10 | 0 | 1 | 0 | 0 | 0 | 5.11E-01 | 3.09E-01 | NA |
| SGSM2 | 0 | 1 | 0 | 0 | 0 | 5.12E-01 | 5.46E-01 | 1.39E-01 |
| NUP155 | 0 | 1 | 1 | 0 | 1 | 5.12E-01 | 1.26E-01 | NA |
| ZNF24 | 0 | 1 | 1 | 0 | 0 | 5.12E-01 | 4.12E-01 | NA |
| POLR2C | 0 | 1 | 1 | 0 | 0 | 5.13E-01 | 2.38E-01 | NA |
| C2orf47 | 0 | 1 | 0 | 0 | 0 | 5.16E-01 | 4.94E-01 | NA |
| NDUFA10 | 0 | 1 | 0 | 0 | 0 | 5.16E-01 | 5.11E-01 | NA |
| RGMA | 0 | 1 | 1 | 0 | 0 | 5.16E-01 | 4.51E-01 | NA |
| REN | 0 | 1 | 0 | 0 | 0 | 5.16E-01 | 2.29E-01 | NA |
| ETV6 | 0 | 1 | 1 | 0 | 0 | 5.20E-01 | 1.95E-01 | NA |
| HIBCH | 0 | 1 | 0 | 0 | 0 | 5.20E-01 | 5.05E-01 | 8.84E-01 |
| LDHAL6B | 0 | 1 | 0 | 0 | 0 | 5.25E-01 | 3.37E-01 | NA |
| PFKP | 0 | 1 | 0 | 0 | 0 | 5.29E-01 | 5.87E-01 | 3.73E-01 |
| TEPP | 0 | 1 | 0 | 0 | 0 | 5.31E-01 | 4.46E-02 | NA |
| NUP214 | 0 | 1 | 0 | 0 | 1 | 5.33E-01 | 3.97E-01 | 7.60E-01 |
| NOA1 | 0 | 1 | 0 | 0 | 0 | 5.35E-01 | 5.64E-01 | 9.04E-02 |
| CDC42EP3 | 0 | 1 | 0 | 0 | 0 | 5.36E-01 | 4.27E-01 | NA |
| SLC35B2 | 0 | 1 | 0 | 0 | 0 | 5.36E-01 | 1.63E-01 | NA |
| HMGCS1 | 0 | 1 | 0 | 0 | 0 | 5.38E-01 | 6.07E-01 | NA |
| VCX2 | 0 | 1 | 0 | 0 | 0 | 5.42E-01 | 7.71E-01 | 8.32E-01 |
| KDM4C | 0 | 1 | 0 | 0 | 0 | 5.43E-01 | 4.79E-01 | NA |
| GCM2 | 0 | 1 | 0 | 0 | 0 | 5.43E-01 | 2.61E-01 | 5.62E-01 |
| SLC35B2 | 1 | 0 | 0 | 0 | 0 | 5.46E-01 | 1.63E-01 | NA |
| HERPUD1 | 0 | 1 | 0 | 0 | 0 | 5.50E-01 | 5.73E-01 | NA |
| GABRA2 | 0 | 1 | 0 | 0 | 0 | 5.53E-01 | 9.75E-03 | NA |
| ZCCHC10 | 0 | 1 | 0 | 0 | 0 | 5.55E-01 | 6.44E-01 | NA |
| MED27 | 0 | 1 | 1 | 1 | 0 | 5.56E-01 | NA | NA |
| MED28 | 0 | 1 | 1 | 1 | 0 | 5.58E-01 | 8.76E-01 | NA |
| SDR39U1 | 0 | 1 | 0 | 0 | 0 | 5.61E-01 | 5.39E-01 | NA |
| OR52L1 | 0 | 1 | 0 | 0 | 0 | 5.64E-01 | 2.37E-01 | 3.31E-01 |
| NUP205 | 0 | 1 | 1 | 0 | 1 | 5.70E-01 | 3.25E-01 | NA |
| PPA1 | 0 | 1 | 0 | 0 | 0 | 5.72E-01 | 4.58E-01 | NA |
| TMPRSS2 | 0 | 1 | 0 | 0 | 0 | 5.72E-01 | 6.82E-01 | 1.06E-01 |
| MGA | 0 | 1 | 0 | 0 | 0 | 5.75E-01 | 4.58E-01 | NA |
| MGLL | 0 | 1 | 0 | 0 | 0 | 5.78E-01 | 6.90E-01 | NA |
| HCFC1 | 0 | 1 | 0 | 0 | 0 | 5.81E-01 | 5.23E-01 | NA |
| NIPSNAP3A | 0 | 1 | 0 | 0 | 0 | 5.82E-01 | 6.30E-01 | 6.22E-01 |
| ATAD3A | 0 | 1 | 0 | 0 | 0 | 5.84E-01 | 6.57E-01 | NA |
| ALDH2 | 0 | 1 | 0 | 0 | 0 | 5.89E-01 | 8.34E-01 | NA |
| OPN1SW | 0 | 1 | 0 | 0 | 0 | 5.90E-01 | 6.64E-01 | NA |
| ZNF519 | 0 | 1 | 0 | 0 | 0 | 5.90E-01 | 5.20E-01 | 7.57E-01 |
| GSTM4 | 0 | 1 | 0 | 0 | 0 | 5.91E-01 | 4.65E-01 | 6.17E-01 |
| SAMD13 | 0 | 1 | 0 | 0 | 0 | 5.92E-01 | 7.32E-01 | NA |
| LAT2 | 0 | 1 | 0 | 0 | 0 | 5.93E-01 | 9.04E-01 | NA |
| SLCO1B1 | 0 | 1 | 0 | 0 | 0 | 5.95E-01 | 6.09E-01 | 6.46E-01 |
| LYSMD2 | 0 | 1 | 1 | 0 | 0 | 6.00E-01 | 6.04E-01 | NA |
| WNK1 | 0 | 1 | 1 | 0 | 0 | 6.01E-01 | 8.04E-01 | 6.98E-01 |
| IK | 0 | 1 | 0 | 0 | 0 | 6.01E-01 | 4.96E-01 | NA |
| OSBPL6 | 0 | 1 | 0 | 0 | 0 | 6.02E-01 | 7.08E-01 | NA |
| MANBA | 0 | 1 | 0 | 0 | 0 | 6.03E-01 | 3.00E-01 | 1.75E-01 |
| POLR2H | 0 | 1 | 1 | 0 | 0 | 6.04E-01 | 3.72E-01 | NA |
| DCUN1D5 | 0 | 1 | 0 | 0 | 0 | 6.06E-01 | 1.89E-01 | NA |
| KCNH3 | 0 | 1 | 0 | 0 | 0 | 6.06E-01 | 1.28E-01 | NA |
| SNRPA | 0 | 1 | 0 | 0 | 0 | 6.08E-01 | 2.84E-02 | NA |
| COQ2 | 0 | 1 | 1 | 0 | 0 | 6.17E-01 | 7.37E-01 | 7.37E-01 |
| NLRX1 | 0 | 1 | 0 | 0 | 0 | 6.17E-01 | 4.28E-01 | 1.22E-01 |
| LRRC30 | 0 | 1 | 0 | 0 | 0 | 6.17E-01 | 4.09E-01 | 7.06E-01 |
| SERTM1 | 0 | 1 | 0 | 0 | 0 | 6.19E-01 | NA | NA |
| KLF12 | 0 | 1 | 0 | 0 | 0 | 6.21E-01 | 7.39E-01 | NA |
| MED31 | 0 | 1 | 1 | 1 | 0 | 6.24E-01 | 6.24E-01 | 6.24E-01 |
| LRP11 | 0 | 1 | 0 | 0 | 0 | 6.27E-01 | 6.59E-01 | NA |
| KBTBD12 | 0 | 1 | 0 | 0 | 0 | 6.27E-01 | 4.32E-01 | NA |
| EIF2A | 0 | 1 | 0 | 0 | 0 | 6.27E-01 | 5.68E-01 | 9.25E-01 |
| CHRFAM7A | 0 | 1 | 0 | 0 | 0 | 6.29E-01 | 5.85E-01 | 5.87E-01 |
| CCDC53 | 0 | 1 | 0 | 0 | 0 | 6.32E-01 | 3.89E-01 | NA |
| SPATA16 | 0 | 1 | 0 | 0 | 0 | 6.35E-01 | 5.93E-01 | NA |
| CORO6 | 0 | 1 | 1 | 0 | 0 | 6.39E-01 | 8.74E-01 | NA |
| RUVBL1 | 0 | 1 | 1 | 0 | 0 | 6.40E-01 | 4.64E-01 | NA |
| ZNF324B | 0 | 1 | 0 | 0 | 0 | 6.42E-01 | 8.81E-01 | NA |
| COX7A2 | 0 | 1 | 0 | 0 | 0 | 6.43E-01 | 4.79E-01 | NA |
| OR4P4 | 0 | 1 | 0 | 0 | 0 | 6.54E-01 | 6.59E-01 | NA |
| TXNL4A | 0 | 1 | 0 | 0 | 0 | 6.56E-01 | NA | NA |
| RNPS1 | 0 | 1 | 0 | 0 | 0 | 6.58E-01 | 6.08E-01 | NA |
| OR10K2 | 0 | 1 | 0 | 0 | 0 | 6.61E-01 | 4.56E-01 | NA |
| CETN3 | 0 | 1 | 0 | 0 | 0 | 6.62E-01 | 7.52E-01 | NA |
| PNMA3 | 0 | 1 | 0 | 0 | 0 | 6.63E-01 | 8.26E-01 | NA |
| ZMAT2 | 0 | 1 | 0 | 0 | 0 | 6.64E-01 | NA | NA |
| COX6B1 | 0 | 1 | 0 | 0 | 0 | 6.64E-01 | 5.95E-01 | NA |
| SLC30A7 | 0 | 1 | 0 | 0 | 0 | 6.65E-01 | 7.11E-01 | NA |
| YDJC | 0 | 1 | 0 | 0 | 0 | 6.65E-01 | 7.86E-01 | NA |
| NUMA1 | 0 | 1 | 0 | 0 | 0 | 6.72E-01 | 7.00E-01 | NA |
| C15orf41 | 0 | 1 | 0 | 0 | 0 | 6.72E-01 | 8.20E-01 | 6.85E-01 |
| MLXIPL | 0 | 1 | 0 | 0 | 0 | 6.74E-01 | 9.65E-01 | NA |
| COG1 | 0 | 1 | 0 | 0 | 0 | 6.74E-01 | 6.61E-01 | NA |
| ISY1 | 0 | 1 | 1 | 0 | 0 | 6.75E-01 | NA | NA |
| SPTSSA | 0 | 1 | 0 | 0 | 0 | 6.76E-01 | NA | NA |
| VWDE | 0 | 1 | 0 | 0 | 0 | 6.78E-01 | 6.54E-01 | 6.04E-01 |
| RANBP2 | 0 | 1 | 1 | 0 | 1 | 6.79E-01 | 7.07E-01 | NA |
| KRTAP19-2 | 0 | 1 | 0 | 0 | 0 | 6.80E-01 | 3.30E-01 | NA |
| ADIPOR1 | 0 | 1 | 0 | 0 | 0 | 6.82E-01 | 9.22E-01 | NA |
| MED10 | 0 | 1 | 0 | 1 | 0 | 6.84E-01 | 8.64E-01 | NA |
| MED14 | 0 | 1 | 1 | 1 | 0 | 6.84E-01 | 5.32E-01 | NA |
| ENO2 | 0 | 1 | 0 | 0 | 0 | 6.85E-01 | 8.29E-01 | NA |
| PLRG1 | 0 | 1 | 0 | 0 | 0 | 6.86E-01 | 5.99E-01 | NA |
| PRPF4 | 0 | 1 | 0 | 0 | 0 | 6.90E-01 | 6.83E-01 | NA |
| MED30 | 0 | 1 | 1 | 1 | 0 | 6.91E-01 | 7.78E-01 | NA |
| SRI | 0 | 1 | 0 | 0 | 0 | 6.93E-01 | 8.58E-01 | NA |
| RNF165 | 0 | 1 | 0 | 0 | 0 | 6.95E-01 | 6.94E-01 | NA |
| FBXL19 | 0 | 1 | 0 | 0 | 0 | 6.95E-01 | 4.10E-01 | NA |
| BRD3 | 0 | 1 | 0 | 0 | 0 | 6.99E-01 | 6.02E-01 | 1.54E-01 |
| ZNF669 | 0 | 1 | 0 | 0 | 0 | 6.99E-01 | 6.49E-01 | NA |
| BAALC | 0 | 1 | 0 | 0 | 0 | 7.02E-01 | 6.99E-01 | 6.97E-01 |
| TRMT10B | 0 | 1 | 0 | 0 | 0 | 7.02E-01 | 4.90E-01 | 7.35E-01 |
| THRAP3 | 0 | 1 | 0 | 0 | 0 | 7.04E-01 | 7.65E-01 | NA |
| BBS10 | 0 | 1 | 1 | 0 | 0 | 7.06E-01 | 5.51E-01 | NA |
| CCDC144A | 0 | 1 | 0 | 0 | 0 | 7.12E-01 | 8.18E-01 | 7.11E-01 |
| TBPL2 | 0 | 1 | 0 | 0 | 0 | 7.15E-01 | 3.23E-01 | NA |
| CSNK1G2 | 0 | 1 | 0 | 0 | 0 | 7.15E-01 | 7.13E-01 | NA |
| EMB | 0 | 1 | 0 | 0 | 0 | 7.17E-01 | 8.30E-01 | 5.81E-01 |
| S100A1 | 0 | 1 | 0 | 0 | 0 | 7.21E-01 | 2.66E-01 | NA |
| CCDC81 | 0 | 1 | 0 | 0 | 0 | 7.22E-01 | 8.78E-01 | 4.29E-01 |
| OXT | 0 | 1 | 0 | 0 | 0 | 7.25E-01 | 7.25E-01 | NA |
| TM9SF3 | 0 | 1 | 0 | 0 | 0 | 7.25E-01 | 6.36E-01 | NA |
| NUP107 | 0 | 1 | 0 | 0 | 1 | 7.26E-01 | 4.26E-01 | NA |
| POLR2G | 0 | 1 | 0 | 0 | 0 | 7.26E-01 | NA | NA |
| ARL9 | 0 | 1 | 0 | 0 | 0 | 7.27E-01 | 6.32E-01 | 6.00E-01 |
| OSMR | 0 | 1 | 0 | 0 | 0 | 7.29E-01 | 5.80E-01 | 7.46E-01 |
| OR13C2 | 0 | 1 | 0 | 0 | 0 | 7.33E-01 | 7.02E-01 | 7.92E-01 |
| PKNOX2 | 0 | 1 | 0 | 0 | 0 | 7.36E-01 | 9.55E-01 | NA |
| TFIP11 | 0 | 1 | 1 | 0 | 0 | 7.37E-01 | 9.41E-01 | NA |
| F13A1 | 0 | 1 | 0 | 0 | 0 | 7.39E-01 | 8.15E-01 | NA |
| NUP93 | 0 | 1 | 0 | 0 | 1 | 7.42E-01 | 6.52E-01 | NA |
| ANKRD6 | 0 | 1 | 0 | 0 | 0 | 7.46E-01 | 6.56E-01 | NA |
| OR2W1 | 0 | 1 | 0 | 0 | 0 | 7.46E-01 | 5.51E-01 | NA |
| EFEMP1 | 0 | 1 | 0 | 0 | 0 | 7.47E-01 | 7.99E-01 | NA |
| PDZD9 | 0 | 1 | 0 | 0 | 0 | 7.49E-01 | 7.82E-01 | 9.33E-01 |
| KRAS | 0 | 1 | 0 | 0 | 0 | 7.51E-01 | NA | NA |
| NHSL1 | 0 | 1 | 0 | 0 | 0 | 7.53E-01 | 6.38E-01 | NA |
| CROCCP3 | 0 | 1 | 0 | 0 | 0 | 7.53E-01 | NA | NA |
| PM20D2 | 0 | 1 | 0 | 0 | 0 | 7.61E-01 | 7.61E-01 | NA |
| EIF4G2 | 0 | 1 | 0 | 0 | 0 | 7.61E-01 | 6.80E-01 | NA |
| SLC9A7 | 0 | 1 | 0 | 0 | 0 | 7.67E-01 | 1.00E+00 | NA |
| MEF2C | 0 | 1 | 0 | 0 | 0 | 7.68E-01 | 7.68E-01 | NA |
| PIGL | 0 | 1 | 0 | 0 | 0 | 7.69E-01 | 7.69E-01 | NA |
| NUDT2 | 0 | 1 | 0 | 0 | 0 | 7.69E-01 | NA | NA |
| MED25 | 0 | 1 | 0 | 1 | 0 | 7.70E-01 | 8.43E-01 | NA |
| RNF5 | 0 | 1 | 0 | 0 | 0 | 7.74E-01 | 7.74E-01 | NA |
| RAB28 | 0 | 1 | 0 | 0 | 0 | 7.77E-01 | 6.97E-01 | NA |
| ARHGAP23 | 0 | 1 | 0 | 0 | 0 | 7.82E-01 | 6.29E-01 | NA |
| CKMT1A | 0 | 1 | 0 | 0 | 0 | 7.84E-01 | 7.11E-01 | NA |
| ATP6V0C | 0 | 1 | 0 | 0 | 0 | 7.87E-01 | NA | NA |
| NIPA2 | 0 | 1 | 0 | 0 | 0 | 7.88E-01 | 8.75E-01 | NA |
| LENG9 | 0 | 1 | 0 | 0 | 0 | 7.89E-01 | 9.72E-01 | 9.63E-01 |
| MED7 | 0 | 1 | 1 | 1 | 0 | 7.92E-01 | 7.85E-01 | NA |
| CPEB4 | 0 | 1 | 0 | 0 | 0 | 8.00E-01 | 6.19E-01 | NA |
| FAM100B | 0 | 1 | 0 | 0 | 0 | 8.01E-01 | 5.44E-01 | NA |
| SEH1L | 0 | 1 | 0 | 0 | 1 | 8.02E-01 | 4.94E-01 | NA |
| NXF1 | 0 | 1 | 0 | 0 | 0 | 8.02E-01 | 6.46E-01 | NA |
| NUP133 | 0 | 1 | 0 | 0 | 1 | 8.03E-01 | 5.96E-01 | NA |
| ERCC5 | 0 | 1 | 0 | 0 | 0 | 8.06E-01 | 7.06E-01 | 1.23E-02 |
| OR2D2 | 0 | 1 | 0 | 0 | 0 | 8.06E-01 | 9.73E-01 | 7.80E-01 |
| CRTAC1 | 0 | 1 | 0 | 0 | 0 | 8.12E-01 | 5.12E-01 | NA |
| CD4 | 1 | 1 | 1 | 0 | 0 | 8.15E-01 | 5.14E-01 | NA |
| ZC3H12A | 0 | 1 | 1 | 0 | 0 | 8.16E-01 | 5.85E-01 | NA |
| NUP88 | 0 | 1 | 0 | 0 | 1 | 8.17E-01 | 8.07E-01 | 1.45E-01 |
| LLPH | 0 | 1 | 0 | 0 | 0 | 8.18E-01 | 6.36E-01 | 7.43E-01 |
| MED24 | 0 | 1 | 0 | 1 | 0 | 8.19E-01 | 3.22E-01 | NA |
| ZNF181 | 0 | 1 | 0 | 0 | 0 | 8.21E-01 | 8.77E-01 | 7.47E-01 |
| RAC1 | 0 | 1 | 0 | 0 | 0 | 8.21E-01 | 7.83E-01 | NA |
| NUP98 | 0 | 1 | 0 | 0 | 1 | 8.21E-01 | 5.23E-01 | NA |
| MED15 | 0 | 1 | 0 | 1 | 0 | 8.27E-01 | 4.14E-01 | NA |
| MED18 | 0 | 1 | 0 | 1 | 0 | 8.27E-01 | 6.65E-01 | NA |
| NUTF2 | 0 | 1 | 1 | 0 | 0 | 8.28E-01 | NA | NA |
| NOL4 | 0 | 1 | 0 | 0 | 0 | 8.28E-01 | 3.59E-01 | NA |
| TCFL5 | 0 | 1 | 0 | 0 | 0 | 8.32E-01 | 8.29E-01 | NA |
| PCDH11X | 0 | 1 | 0 | 0 | 0 | 8.33E-01 | 8.38E-01 | NA |
| LGALS9 | 0 | 1 | 0 | 0 | 0 | 8.34E-01 | 8.82E-01 | NA |
| MED23 | 0 | 1 | 0 | 1 | 0 | 8.35E-01 | 7.55E-01 | NA |
| ZNF576 | 0 | 1 | 1 | 0 | 0 | 8.36E-01 | 8.19E-01 | NA |
| DDX19B | 0 | 1 | 0 | 0 | 0 | 8.36E-01 | 9.10E-01 | NA |
| HIST1H3I | 0 | 1 | 0 | 0 | 0 | 8.38E-01 | 7.42E-01 | 7.42E-01 |
| RPS10 | 0 | 1 | 0 | 0 | 0 | 8.40E-01 | NA | NA |
| AIFM3 | 0 | 1 | 0 | 0 | 0 | 8.41E-01 | 6.74E-01 | NA |
| ITLN1 | 0 | 1 | 1 | 0 | 0 | 8.42E-01 | 4.79E-01 | NA |
| IGFN1 | 0 | 1 | 1 | 0 | 0 | 8.42E-01 | 8.62E-01 | 1.76E-01 |
| POLR2F | 0 | 1 | 0 | 0 | 0 | 8.43E-01 | 7.74E-01 | NA |
| NAT8L | 0 | 1 | 0 | 0 | 0 | 8.46E-01 | NA | NA |
| POLR2A | 0 | 1 | 0 | 0 | 0 | 8.48E-01 | 7.65E-01 | NA |
| XKR4 | 0 | 1 | 0 | 0 | 0 | 8.49E-01 | 8.63E-01 | NA |
| FAS | 0 | 1 | 0 | 0 | 0 | 8.49E-01 | 8.61E-01 | NA |
| SPARC | 0 | 1 | 0 | 0 | 0 | 8.51E-01 | 7.40E-01 | NA |
| OR10J1 | 0 | 1 | 0 | 0 | 0 | 8.55E-01 | 8.72E-01 | 9.86E-01 |
| SPATA3 | 0 | 1 | 0 | 0 | 0 | 8.56E-01 | 5.93E-01 | NA |
| GSC | 0 | 1 | 0 | 0 | 0 | 8.58E-01 | NA | NA |
| FAM71C | 0 | 1 | 0 | 0 | 0 | 8.62E-01 | 6.11E-01 | 4.80E-01 |
| NUP43 | 0 | 1 | 0 | 0 | 1 | 8.64E-01 | 9.12E-01 | 8.29E-01 |
| COG3 | 0 | 1 | 0 | 0 | 0 | 8.67E-01 | 5.99E-01 | NA |
| RGPD4 | 0 | 1 | 1 | 0 | 0 | 8.67E-01 | 6.72E-01 | 4.04E-01 |
| SFR1 | 0 | 1 | 1 | 0 | 0 | 8.67E-01 | 3.60E-01 | NA |
| COL14A1 | 0 | 1 | 0 | 0 | 0 | 8.72E-01 | 8.63E-01 | 1.20E-01 |
| COG4 | 0 | 1 | 0 | 0 | 0 | 8.72E-01 | 6.85E-01 | 7.49E-01 |
| MED29 | 0 | 1 | 0 | 1 | 0 | 8.72E-01 | 8.74E-02 | NA |
| TSC22D2 | 0 | 1 | 1 | 0 | 0 | 8.75E-01 | 4.24E-01 | NA |
| LINC00346 | 0 | 1 | 0 | 0 | 0 | 8.76E-01 | 6.27E-01 | 2.95E-01 |
| MED26 | 0 | 1 | 0 | 1 | 0 | 8.81E-01 | 7.93E-01 | NA |
| RPL27A | 0 | 1 | 0 | 0 | 0 | 8.83E-01 | 4.08E-02 | NA |
| RAE1 | 0 | 1 | 0 | 0 | 1 | 8.83E-01 | 7.42E-01 | NA |
| CS | 0 | 1 | 1 | 0 | 0 | 8.84E-01 | 1.48E-01 | NA |
| LILRA3 | 0 | 1 | 0 | 0 | 0 | 8.84E-01 | 8.59E-01 | 4.37E-01 |
| SPAG6 | 0 | 1 | 1 | 0 | 0 | 8.85E-01 | 3.63E-01 | 7.37E-01 |
| C11orf35 | 0 | 1 | 0 | 0 | 0 | 8.88E-01 | 6.65E-01 | NA |
| OR2A5 | 0 | 1 | 0 | 0 | 0 | 8.88E-01 | 8.97E-01 | 6.28E-01 |
| CCDC59 | 0 | 1 | 0 | 0 | 0 | 8.89E-01 | 9.05E-01 | 1.45E-01 |
| ARCN1 | 0 | 1 | 0 | 0 | 0 | 8.92E-01 | 8.53E-01 | NA |
| AAAS | 0 | 1 | 0 | 0 | 1 | 8.92E-01 | 8.74E-01 | 6.40E-01 |
| KDELR2 | 0 | 1 | 1 | 0 | 0 | 8.92E-01 | 6.11E-01 | NA |
| AHCTF1 | 0 | 1 | 1 | 0 | 1 | 8.93E-01 | 6.84E-01 | NA |
| POLR2E | 0 | 1 | 0 | 0 | 0 | 8.97E-01 | 1.18E-01 | NA |
| POLR2L | 0 | 1 | 1 | 0 | 0 | 9.01E-01 | 7.64E-01 | NA |
| DEFB131 | 0 | 1 | 0 | 0 | 0 | 9.05E-01 | 9.05E-01 | NA |
| CD8B | 0 | 1 | 0 | 0 | 0 | 9.12E-01 | 4.06E-01 | NA |
| BCKDK | 0 | 1 | 0 | 0 | 0 | 9.26E-01 | 8.48E-01 | 7.33E-01 |
| C9orf53 | 0 | 1 | 1 | 0 | 0 | 9.33E-01 | 3.51E-01 | NA |
| TMPRSS7 | 0 | 1 | 0 | 0 | 0 | 9.34E-01 | 9.62E-01 | 7.42E-01 |
| BOLA3 | 0 | 1 | 1 | 0 | 0 | 9.35E-01 | 8.85E-01 | NA |
| OCM2 | 0 | 1 | 0 | 0 | 0 | 9.35E-01 | 6.66E-01 | 6.66E-01 |
| RPL26L1 | 0 | 1 | 0 | 0 | 0 | 9.37E-01 | NA | NA |
| MTF1 | 0 | 1 | 0 | 0 | 0 | 9.41E-01 | 9.29E-01 | NA |
| LARS | 0 | 1 | 1 | 0 | 0 | 9.49E-01 | 9.29E-01 | 6.79E-01 |
| FAM182A | 0 | 1 | 0 | 0 | 0 | 9.53E-01 | 9.53E-01 | NA |
| ARID3C | 0 | 1 | 1 | 0 | 0 | 9.59E-01 | 9.90E-01 | NA |
| ALS2CR12 | 0 | 1 | 0 | 0 | 0 | 9.60E-01 | 9.61E-01 | 8.53E-01 |
| TBL2 | 0 | 1 | 0 | 0 | 0 | 9.64E-01 | 9.11E-01 | NA |
| C1D | 0 | 1 | 0 | 0 | 0 | 9.64E-01 | 5.49E-01 | NA |
| MORC1 | 0 | 1 | 0 | 0 | 0 | 9.72E-01 | 9.68E-01 | NA |
| EQTN | 0 | 1 | 0 | 0 | 0 | 9.72E-01 | 9.68E-01 | NA |
| RBM44 | 0 | 1 | 0 | 0 | 0 | 9.72E-01 | 9.18E-01 | 6.37E-01 |
| INVS | 0 | 1 | 0 | 0 | 0 | 9.75E-01 | 9.73E-01 | NA |
| NCAPG | 0 | 1 | 0 | 0 | 0 | 9.76E-01 | 9.62E-01 | 5.42E-01 |
| OR10G2 | 0 | 1 | 0 | 0 | 0 | 9.77E-01 | 9.07E-01 | NA |
| TCF21 | 0 | 1 | 0 | 0 | 0 | 9.88E-01 | 8.82E-01 | NA |
| STX5 | 0 | 1 | 1 | 0 | 0 | 9.89E-01 | 9.69E-01 | 6.79E-01 |
| RFPL3 | 0 | 1 | 0 | 0 | 0 | 9.91E-01 | 8.64E-01 | NA |
| PRRC2B | 0 | 1 | 0 | 0 | 0 | 9.91E-01 | 9.63E-01 | NA |
| ENKD1 | 0 | 1 | 1 | 0 | 0 | 9.92E-01 | 9.96E-01 | NA |
| PRPF18 | 0 | 1 | 0 | 0 | 0 | 9.96E-01 | 9.93E-01 | NA |
| DDX60L | 0 | 1 | 0 | 0 | 0 | 9.98E-01 | 9.91E-01 | 9.24E-01 |
| NAPSA | 0 | 1 | 0 | 0 | 0 | >9.99E-01 | 9.96E-01 | 7.22E-01 |

^a^Digits indicate whether a gene was included (1) or excluded (0) as part of the set

^b^NA indicates that no variants of that class were observed in that gene
