# Supplementary material for: Evaluating the Impact of Functional Genetic Variation on HIV-1 Control
Source: J Infect Dis. 2017 Sep 9;216(9):1063–9. doi: 10.1093/infdis/jix470 (PMC5853944; doi:10.1093/infdis/jix470)
Supplement: Supplementary Table S4 [file jix470_suppl_supplementary_table_s4.docx]

**Table S4: Linkage between rs1055821 and variants previously linked to HIV control**

| **Variant** | **R2** | **D'** | **Reference** |
| --- | --- | --- | --- |
| rs2395029 | 0.55 | 1.0 | Fellay et al, Science 2007; Pereyra et al Science 2010 |
| rs9264942 | 0.09 | 0.78 | Fellay et al, Science 2007; Pereyra et al Science 2010 |
| HLA-B pos97(Val) | 0.6 | 1.0 | Pereyra et al Science 2010 |
